# White matter inflammation and cognitive function in a co-morbid metabolic syndrome and prodromal Alzheimer’s disease rat model

**DOI:** 10.1186/s12974-020-1698-7

**Published:** 2020-01-21

**Authors:** Nadezda Ivanova, Qingfan Liu, Cansu Agca, Yuksel Agca, Earl G. Noble, Shawn Narain Whitehead, David Floyd Cechetto

**Affiliations:** 1 Department of Anatomy & Cell Biology, Schulich School of Medicine & Dentistry, Western University, London, ON N6A 5C1 Canada; 2 Department of Veterinary Pathobiology, University of Missouri College of Veterinary Medicine, Columbia, MO USA; 3 School of Kinesiology, Western University, London, ON Canada

**Keywords:** Prodromal Alzheimer’s disease, Metabolic syndrome, Hypercaloric diet, APP21 Transgenic rat, White matter inflammation, Microglia

## Abstract

**Background:**

Metabolic syndrome, the development of which is associated with high-caloric Western diet (HCD) intake, represent a risk factor for mild cognitive impairment (MCI) and dementia including Alzheimer’s disease (AD) later in life. This study aimed to investigate the effect of diet-induced metabolic disturbances on white matter neuroinflammation and cognitive function in a transgenic (TG) Fischer 344 rat carrying a human β-amyloid precursor protein (APP) gene with Swedish and Indiana mutations (APP21 TG), a model of pre-AD and MCI.

**Methods:**

TG and wildtype (WT) rats received either a HCD with 40% kJ from fat supplemented with 20% corn syrup drink or a standard diet for 12 weeks. Body weight, caloric intake, and blood pressure were measured repeatedly. End-point changes in glucose and lipid metabolism were also assessed. Open field task was used for assessment of activity; Morris water maze was used to assess spatial learning and memory. Cerebral white matter microglia and astrocytes, hippocampal neurons, and neuronal synapses were examined using immunohistochemistry.

**Results:**

Rats maintained on the HCD developed significant obesity, visceral adiposity, dyslipidemia, and hyperinsulinemia, but did not become hypertensive. Impaired glucose tolerance was observed only in WT rats on the HCD. Total microglia number, activated OX-6+ microglia, as well as GFAP+ astrocytes located predominantly in the white matter were greater in the APP21 TG rat model in comparison to WT rats. HCD-driven metabolic perturbations further exacerbated white matter microgliosis and microglia cell activation in the APP21 TG rats and led to detectable changes in spatial reference memory in the comorbid prodromal AD and metabolic syndrome group compared to WT control rats. Neuronal density in the CA1 subregion of the hippocampus was not different between the experimental groups. Synaptic density in the CA1 and CA3 hippocampal subregions was lower in the TG rats compared to WT rats; however, there was no additional effect of the co-morbidity on this measure.

**Conclusions:**

These results suggest that white matter neuroinflammation might be one of the possible processes of early interaction of metabolic syndrome with MCI and pre-AD and could be one of the early brain pathologies contributing to cognitive deficits observed in mild cognitive impairment and dementia, including AD cases.

## Background

Among age-related diseases, dementias are particularly serious given their prevalence, severity, and progressive and incurable characteristics. Alzheimer’s disease (AD) is the most commonly diagnosed form of dementia. The accumulation of amyloid-β peptide (Aβ), produced through an altered cleavage of amyloid precursor protein (APP), and formation of neurofibrillary tangles are considered to be the hallmarks of AD [1]. The processes associated with the development of AD include glial activation, excessive neuroinflammation, and oxidative stress, as well as vascular and metabolic abnormalities [1, 2]. AD is a disease impacting both the gray and white matter of the brain. While changes to the gray matter in the pathogenesis of AD are well known and continued to be heavily investigated, the neuropathology of white matter abnormalities still remains not fully understood and is mainly attributed to cerebral small vessel degeneration, inflammatory events, as well as loss of myelin and axonal fibers [3–6]. However, white matter changes have been shown to develop very early, in prodromal phase (pre-AD) and precede the onset of clinical symptoms of dementia, highlighting the importance of their further investigation [7, 8].

The complex etiology and pathology of AD alone remains a focus of research, but increasing attention is paid to the interplay of AD with comorbidities such as stroke and metabolic disorders including diabetes and metabolic syndrome [9]. Metabolic syndrome, which is a focus of our research work, represents a combination of conditions such as obesity, dyslipidemia, glucose intolerance, insulin resistance, and hypertension. Unhealthy lifestyle choices play a big role in etiology of metabolic syndrome, with chronic intake of high calorie Western diets rich in saturated fat and simple carbohydrates coupled with a sedentary lifestyle being the most common risk factors [10, 11]. Metabolic syndrome is a serious public health issue [12]. It begins in middle age and continues to develop over time manifesting in serious conditions such as type 2 diabetes, cardio- and cerebrovascular diseases. Moreover, it represents a risk factor for dementia, including AD [13–16], and often coexist with it in one individual likely contributing to the course and progression of dementia [17].

Epidemiological and clinical studies strongly suggest the existence of an interaction between metabolic syndrome and dementia, including mild cognitive impairment (MCI) and AD. Individuals obese and diagnosed with metabolic syndrome show a greater risk for developing cognitive decline later in life [18–20] and AD patients tend to have a poorer prognosis when metabolic syndrome is also present [21]. Experimental data from studies using rodent models of well-developed AD fed a high-fat diet yield evidence of poor performance in cognitive tasks and increased AD-like pathology including neuroinflammation [22–26]. In contrast to the earlier studies, our present study aimed to examine the early processes and interactions occurring at the prodromal phase of AD using a novel transgenic model of high cerebral amyloid levels as a predisposing environment.

Inflammation as an event associated with both dementia, including AD, and metabolic syndrome has been suggested to be one of the shared mechanisms contributing to the impaired cognition and AD-like pathology [27–29]. In the current study, we examined the early effects of the comorbidity on the inflammation in the white matter which is highly susceptible to pathological changes, particularly the key cellular components of inflammatory response, microglia and astrocytes.

While there is a clear connection between metabolic diseases and AD, the exact underlying mechanisms regarding how metabolic diseases affect mental health and contribute to the existing neuropathology, especially at the very initial stages of their development, remain unclear. The gap in our understanding of this interaction appears to be a limiting factor in any success in finding effective therapeutic and preventive interventions. This highlights the importance of developing experimental models that combine prodromal phase AD-like pathology with risk factors such as metabolic syndrome to investigate the potential of early intervention and prevention.

The present study was undertaken to better understand the relation between metabolic abnormalities and prodromal AD dementia, particularly studying the impact on changes in white matter inflammatory pathology and coincident cognitive deficits. The comorbidity of prodromal AD with metabolic syndrome was examined in a novel APP21 transgenic (TG) rat model of pre-AD [30, 31] created on a Fischer 344 background which carries a human APP (hAPP) gene with Swedish and Indiana mutations, implicated in early-onset AD. This rat has been previously shown to express high levels of human brain APP and serum β-amyloid (Aβ1-40 and 1-42) without spontaneous Aβ plaques deposition in brain tissue with age [32, 33]. Thus, it allows us to study the early interaction between metabolic syndrome and prodromal AD-like processes in the brain in a model with AD-predisposing conditions.

In this study we focused on the pathology of diet-induced metabolic syndrome in relation to prodromal phase of AD, specifically examining the consequences of its chronic course on the white matter inflammation, one of the earliest and most critical events occurring in the brain in response to insult, particularly on its key cellular players, microglia, and astrocytes. In addition, we examined the effects of diet in the pre-AD model on behavior and cognitive function. The hypothesis is that there would be greater white matter inflammation and cognitive deficits in the combined model than in either condition alone.

## Methods

### Animals

**Fig. 1:**
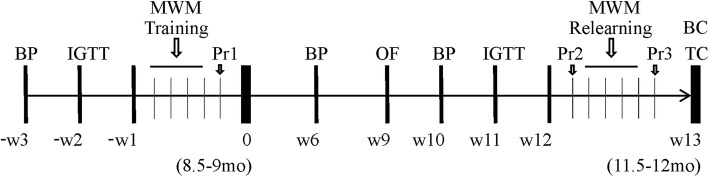
Project timeline. Rat’s age (in months) at the start (day 0) and the end (week 13) of the study are shown in brackets. Diets were assigned on day 0 and all testing time points are in reference to this day. Baseline measurements were completed 3 weeks prior to the start of the diet. Morris water maze spatial training was completed on week − 1 (4 days, four trials a day) with a probe trial (Pr1) following on the day after. A second probe trial (Pr2) was completed on week 12 on a diet. Learning of a new platform location started the next day following the same protocol with a probe trial (Pr3) at the end. *BP* blood pressure measurement, *IGTT* intraperitoneal glucose tolerance test, *MWM* Morris Water Maze, *Pr* probe trial, *OF* open field test, *BC* blood collection, *TC* tissue collection, *W* week

All animal handling and experimental procedures were approved by Western University Animal Care Committee (AUP 2008-113) and were carried out in accordance with the guidelines of the Canadian Council on Animal Care and National Institute of Health Guides for the Care and Use of Laboratory Animals. A total of 24 male wildtype (WT) and 22 male APP21 TG Fischer 344 rats were involved in this study, and rats were assigned to experimental groups at random. Rats were bred in-house with original breeding pairs obtained from Drs. Yuksel Agca and Cansu Agca (University of Missouri, Colombia, MO, USA) [30] and confirmed to be homozygous. Animals were housed in pairs under standard conditions (12:12 light/dark cycle, at 22–24 °C) and maintained on a standard rat diet provided ad libitum. At the age of 8.5–9.5 months, half of the rats of each genotype were randomly assigned to a high-calorie Western type diet (HCD), while the other half continued on a standard diet (Control diet, CD). Diets were provided ad libitum and rats were maintained on the diets for 12 weeks. A study timeline is shown in Fig. 1. Body weight as well as food and drink consumption were measured twice a week throughout the experiment. Toward the end of the experiment, there were slight variations in the exact time for the physiological and metabolic measures since they would interfere with the acquisition of behavioral data. Animal numbers for each experimental dietary group were as follows: Control WT, *n* = 12; Control TG, *n* = 11; HCD WT, *n* = 12; and HCD TG, *n* = 11.

### Diets

Rats maintained on a standard diet received chow with the following composition (in %kJ): 26 protein, 59.7 carbohydrate, and 14.3 fat with 1.52 % of saturated fatty acid (Prolab RMH 3000 5P00). The Western diet consisted of the following (in %kJ): 17 protein, 43 carbohydrate, and 40 fat with 62.4% of saturated fatty acid (D12079B, Research Diets, Inc) which included 0.21% cholesterol. The metabolizable energy from standard and Western diet (in kJ/g) was 13.31 and 19.66, respectively. The solid food was supplemented with water in the CD group and with 20% corn syrup water solution in the HCD group as an additional source of calories (Bee Hive, ACH Food Companies, Inc, USA).

### Intraperitoneal glucose tolerance test and insulin measurement

Intraperitoneal glucose tolerance test (IGTT) was performed at 2 weeks prior, and 11 weeks following the change in diet (Fig. 1). Following a 12-h overnight fast, 100–150 μl of blood was drawn from the saphenous vein for determination of glucose and insulin baseline levels. A 60% glucose solution in 0.9% saline (2 g/kg) was then injected intraperitoneally. Blood was collected from a tail vein repeatedly at 15, 30, 60, 90, and 120 min after the glucose load. Glucose levels (mmol/l) were measured using Freestyle Light Blood Glucose Monitoring System (Abbott Diabetes Care Inc, Alameda, CA). Glucose responses over time were analyzed to determine the area-under-the-curve (AUC). Fasting insulin levels were determined in serum samples using an ELISA kit (Ultra-sensitive rat insulin ELISA kit, Crystal Chem. Inc) according to the manufacturer’s instructions. Homeostasis model assessment index (HOMA) was calculated to estimate insulin resistance using the following formula [34]: HOMA-IR = (fasting glucose (mmol/l) × fasting insulin (mmol/l)/22.5.

### Lipid profile analysis

Triglycerides, total cholesterol, and high-density lipoprotein (HDL) cholesterol were measured in serum samples isolated from cardiac blood at time of euthanasia and analyzed at the Clinical Laboratory at University Hospital (London, ON, Canada). Non-HDL cholesterol was calculated as total cholesterol − HDL cholesterol. The cholesterol ratio (Chol:HDL ratio) was calculated by dividing total cholesterol value by HDL number.

### Blood pressure analysis

Systolic and diastolic arterial blood pressure were assessed 3 weeks before and at weeks 6 and 10 on the diet (Additional file 1: Figure S1) via a non-invasive tail cuff method (CODA Blood Pressure System, Kent Scientific Corp., Connecticut, USA).

### Open field activity and anxiety-like behavior

Locomotor activity and anxiety were tested in a square open field arena (Med Associates Inc., St. Albans, VT, USA) over the course of 20 min on week 9 of the diet (Fig. 1). Ambulatory distance and time spent in central and peripheral zones were evaluated using Activity Monitor software, Med Associates Inc.

### Morris water maze

Rats first encountered the Morris water maze test (MWM) 1 week prior to the diet onset. The second testing (relearning) was performed 12 weeks after the diet following the same protocol, but with a new platform location (Fig. 1). Rats were trained to find a hidden escape platform in a circular pool (145 cm in diameter, 58 cm in depth) filled with water, dyed with black non-toxic acrylic paint, using extra-maze cues placed on the walls around the pool. The training protocol consisted of 16 trials over four consecutive days (four trials/day). The duration of one trial was 60 s with a 30-s inter-trial period during which time the rats remained on the platform. The platform (12 cm in diameter) was placed in the middle of one of four virtual quadrants the pool was divided into, and this location remained unchanged during the training phase. Start positions were presented in a randomized order for every day of spatial acquisition. Learning progress was assessed using time and distance required to reach the platform and path efficiency (ratio of direct path length to the platform to actual path length, 1 being most efficient) in the acquisition trials. The day after the last day of training, the rats were subjected to a 30-s probe trial where the platform was removed from the pool and the rats were released from a novel start position. At the end of the training and probe prior to the dietary manipulation, two reacquisition trials in which the platform was returned to the previous position were administered to prevent memory extinction. Performance was evaluated using such parameters as time and distance traveled in the quadrant of a previous platform location (target quadrant) and swimming speed. Performance was monitored using video-tracking software (ANY-maze®, Stoelting Co., Wood Dale, IL, USA).

### Euthanasia and tissue collection

Following a 12-h fasting period, the rats were weighed and euthanized by a pentobarbital overdose. Cardiac blood was collected immediately before perfusion. Epididymal fat pads were collected and weighed. Rats were then perfused transcardially with 0.01 M phosphate-buffered saline (pH 7.35) followed by 4% paraformaldehyde (PFA, pH 7.35). Brains were post-fixed in PFA overnight and then transferred to a 30% sucrose solution until saturated fully submerged. Brains were sectioned coronally on a cryostat into 35-μm-thick sections approximately from bregma 4.70 mm to bregma − 5.20 mm [35], sorted into 12 series and were stored in cryoprotectant at − 20 °C until used for immunohistochemistry.

### Immunohistochemistry

Immunohistochemistry was performed on free-floating sections to visualize microglia, activated microglia, astrocytes, neurons, synapses, and human amyloid-β accumulates using rabbit polyclonal antibodies against the ionized calcium binding adaptor molecule-1 (anti-Iba-1; 1:1000; Wako Chemicals USA Inc., Richmond, VA, USA), inducible nitric oxide synthase (anti-iNOS; 1:1000; Abcam Inc, Toronto, ON, Canada), and mannose receptor (anti-cluster of differentiation CD206; 1:200; Abcam Inc, Toronto, ON, Canada); mouse monoclonal antibodies directed against the major histocompatibility complex II (MHC II) receptor (OX-6; 1:1000; BD Pharmingen, Mississauga ON, Canada), glial acidic fibrillary protein (anti-GFAP; 1:2000; Sigma-Aldrich, St Louis MO, USA), neuronal nuclei (anti-NeuN; 1:1000; EMD Millipore Corp., USA), synaptophysin, a major synaptic vesicle protein, (anti-synaptophysin; 1:1000; Sigma-Aldrich, St Louis MO, USA), and amino acid residues 17-24 of the amyloid-β (anti-β-amyloid 4G8; 1:500; BioLegend, San Diego CA, USA), respectively. Antigen retrieval was performed prior to primary antibody incubations for anti-iNOS and anti-amyloid staining with citric acid buffer at 95 °C and 70% formic acid, respectively. Following an overnight incubation with the primary antibody at 4 °C, sections were incubated with biotinylated anti-mouse or anti-rabbit secondary antibody (1:500, 1:1000 (iNOS) or 1:10000 (CD206), Vector Laboratories, Inc. Burlingame, CA, USA) followed by incubation with avidin-biotin complex (ABC kit, Vector Laboratories, Inc. Burlingame, CA, USA) reagent and then developed in 0.05% 3,3′diaminobenzidine tetrahydrochloride (Sigma-Aldrich, St. Louis MO, USA). Sections were then mounted on glass slides, air-dried, dehydrated, cleared in xylene, and coverslipped with DePex mounting media (DePex, BDH Chemicals, Poole, UK). Detection of changes in white matter fiber myelination was done in sections pre-washed in 0.01 M PBS mounted on glass slides, dried overnight, and stained with Luxol fast blue following the protocol described elsewhere [36].

### Imaging and quantification of immunohistochemistry

Immunohistochemically and histochemically processed brain sections were imaged at × 10 objective with a Nikon Eclipse Ni-E upright microscope with a Nikon DS Fi2 color camera head using NIS-Elements Imaging Software Version 4.30.02 (Nikon Instruments Inc., Melville, NY). Brain sections stained for OX-6 and Luxol fast blue were scanned with Aperio digital entire-slide scanner, allowing × 20 magnification (Department of Pathology, Western University, London, Ontario, Canada). Entire series of brain sections was screened for positive OX-6 signal to determine regions of interest (ROIs) for all further analysis. Analysis and quantification were carried out using 64-bit ImageJ software (Version 1.48u4, Wayne Rasband, National Institutes of Health, Bethesda, MD, USA). The investigator was blinded to the identity of rats included in the quantification analysis. Images were converted into a black-and-white 8-bit format, underwent thresholding, and were calibrated prior to taking all the measurements. Based on the location of the positive OX-6 immunostaining being mainly in the white matter structures, the corpus callosum, internal capsule, and fimbria of the dorsal hippocampi were chosen as ROIs. A total of six regions from three consecutive brain sections containing corpus callosum, internal capsule, or fimbria were analyzed for each animal. For the assessment of activated microglia cells (OX-6 stained) in the corpus callosum and internal capsule, areas with positive signal were manually outlined using a free outline tool. Integrated density, defined as a sum of the values of the pixels in the selected area, was measured for each region and summarized into a single value per animal. To analyze changes in general microglia population (Iba-1stained), activation of astrocytes, activated microglia in the fimbria and myelin content, white matter tracts were manually outlined, and a measure of the area of coverage by positive signal (percent of the total area) was noted for each region and expressed as a weighted average. Additionally, automated cell counting was done for OX-6, Iba-1, and GFAP stained glia in each ROI. An average number of cells per 100 mm^2^ of each ROI per rat was determined using ImageJ particle analysis. The neuronal population of the hippocampus, CA1 subregion, was visualized with NeuN immunostaining and was assessed using the NIS Elements analysis software. In the ROI sampled from two to three coronal brain sections, neuronal nuclei were automatically counted in a selected field of 0.2 mm^2^ area and an average number was generated for each animal. Synaptophysin staining was quantified in the CA1 and CA3 hippocampal subregions in a total of eight fields per subregion, sampled from two brain sections, per animal in the ImageJ. The area of coverage by positive signal was expressed as a weighted average.

### Data analysis

Statistical analysis was performed using GraphPad Prism 6.0. Data were analyzed by performing *t* test or one-way or two-way analysis of variance (ANOVA), followed by Tukey’s multiple comparisons test. Data is expressed as mean ± standard error of the mean (SEM), and a *p* value of ≤ 0.05 was considered statistically significant.

## Results

First, we performed an extensive physiological characterization of the model by analyzing body weights, parameters of glucose and lipid metabolism, and arterial blood pressure values.

### Body weights, diet, fat accumulation, lipid and glucose metabolism

**Fig. 2:**
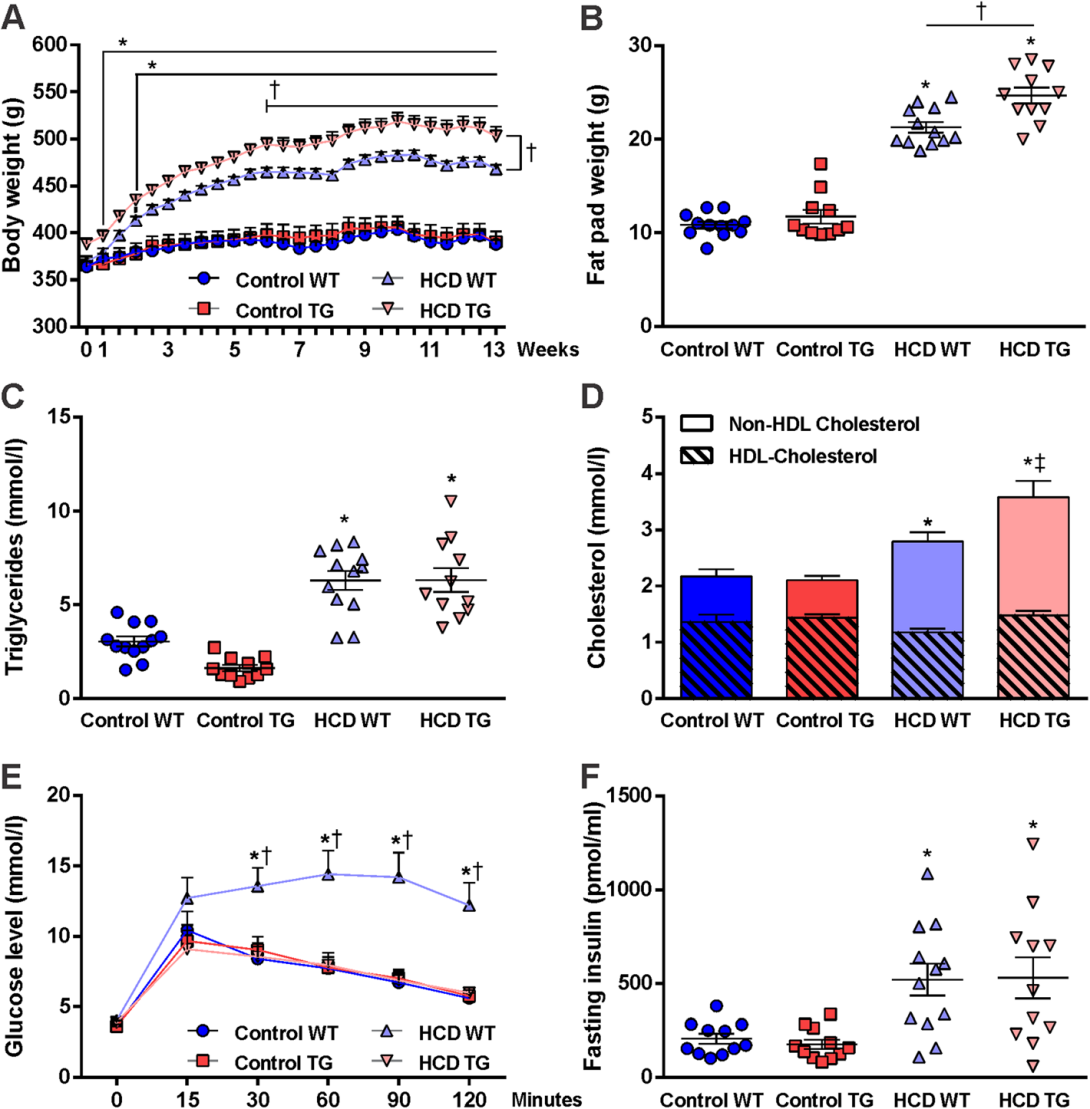
Weight gain, visceral fat accumulation, lipid, and glucose metabolism*.* **a** Body weight change over the course of the diets. **b** Post-mortem paired epididymal fat pad weight. **c** Fasting triglyceride levels measured at the end of week 12 on the diets. **b** Fasting levels of total cholesterol presented as the whole bar and its fractions: Non-HDL (upper part of a bar) and HDL-Cholesterol (lower part of a bar). **e** Blood glucose levels during 2-h intraperitoneal glucose tolerance test (IGTT) after 11 weeks on the diets. Zero time point (0) represents fasting glucose value obtained immediately before glucose load. **f** Fasting insulin levels measured from a blood sample drawn at time point 0 during IGTT. Animal numbers are as follows: control WT (*n* = 12), control TG (*n* = 11), HCD WT (*n* = 12), HCD TG (*n* = 11). Values are presented as mean ± SEM. Significance is indicated by * between HCD and both control groups (in **d**—for non-HDL cholesterol), † between HCD groups, and ‡ between HCD TG and both control groups for total cholesterol. RM two-way ANOVA and one-way ANOVA, Tukey’s multiple comparisons test, *p* < 0.05. *HCD* hypercaloric diet, *HDL* high-density lipoprotein, *TG* transgenic, *WT* wildtype

Both TG and WT rats on a HCD gained weight rapidly and weighed significantly more than CD groups as early as the first week on the diet (Fig. 2a). Starting from week 6 on the diet, rats from the comorbid group weighed more than the HCD WT group, and this weight difference remained significant until the end of the study. In addition, as shown in Fig. 2b, epididymal fat pads mass was significantly increased with HCD consumption (*F*_(1,42)_ = 335.9; *p* < 0.0001), with an even greater increase in the TG rats (genotype effect *F*_(1,42)_ = 11.26; *p* < 0.0017, interaction *F*_(1,42)_ = 3.769; *p* < 0.059). Analysis of diet consumption across the 12 weeks showed a decrease in amount of food consumed (Control WT 20 ± 0.2, Control TG 21 ± 0.3 vs. HCD WT 9 ± 0.2, HCD TG 11 ± 0.4 g/day); however, there was a large increase in drink consumption by rats from both HCD groups (control WT 23 ± 0.7, control TG 27 ± 0.7 vs. HCD WT 68 ± 2.4, HCD TG 61 ± 1.9 g/day). This resulted in a significantly higher total energy intake in the HCD groups during the entire period of 12 weeks (control WT 264 ± 3.0, control TG 272 ± 4.0 vs. HCD WT 406 ± 5.0, HCD TG 402 ± 3.0 kJ/day). Based on genetic profiles, rats had different preferences for the source of calories; TG rats favored high-fat food, whereas WT rats had a stronger preference for carbohydrates from drink. Triglyceride levels were significantly elevated by HCD (Fig. 2c).

Total cholesterol was increased in both groups on the HCD, but reached statistical significance only in the comorbid rats compared to controls. Both TG and WT rats on the HCD had an increased cholesterol content of atherogenic lipoprotein particles (non-HDL cholesterol; Fig. 2d). HDL cholesterol levels, when analyzed separately, were not different for HCD rats in comparison to the control groups. However, the Chol:HDL ratio, a relevant clinical index, was significantly greater in both HCD groups, indicating that these rats had a decrease in HDL cholesterol and a significant shift toward the atherogenic Non-HDL fraction (Table 1).

**Table 1:** Serum glucose-, insulin-, and lipid-related measures

	Control WT	Control TG	HCD WT	HCD TG
Glucose, mmol/l (initial)	3.73 ± 0.10	3.82 ± 0.07		
Glucose, mmol/l (post)	3.6 ± 0.13	3.63 ± 0.14	3.96 ± 0.13	3.91 ± 0.12
AUC IGTT (initial)	929.98 ± 45.91	786.08 ± 53.51		
AUC IGTT (post)	891.08 ± 82.09	907.25 ± 79.11	1568.85 ± 172.74^*†^	892.44 ± 90.58
Insulin, pmol/ml (post)	243.1 ± 28.60	237.9 ± 29.16		
HOMA-IR (post)	0.19 ± 0.03	0.17 ± 0.03	0.56 ± 0.09^*^	0.58 ± 0.13^*^
Chol:HDL, mmol/l	1.64 ± 0.09	1.49 ± 0.06	2.43 ± 0.17^*^	2.44 ± 0.17^*^

Initial—data obtained prior to the diet assignment; rats used in the study were combined by genotype. Post—data collected after 11–12 weeks on the diets. Non-specified—data obtained at the end of the study. All measures (except AUC IGTT) represent fasting state values. Values are presented as mean SEM. The symbols * and † indicate significance for HCD group vs. both control groups and between HCD groups respectively. One-way ANOVA, Tukey’s multiple comparison test; *p* < 0.05. *AUC* area-under-the-curve, *Chol* total cholesterol, *HCD* hypercaloric diet, *HDL* high-density lipoprotein cholesterol, *HOMA*-*IR* homeostasis model assessment index, *IGTT* intraperitoneal glucose tolerance test, *TG* transgenic, *WT* wildtype

### Glucose metabolism

Rats maintained on HCD did not show signs of hyperglycemia based on the fasting blood levels of glucose (Table 1). Surprisingly, a glucose intolerance pattern was observed only in WT rats maintained on a HCD, which was characterized by a greater increase in blood glucose levels at 30 min after a glucose injection that remained significantly increased till the end of a 2-h period (Fig. 2e). This also translated into a significantly greater AUC for blood glucose. There appeared to be no effect of diet on glucose tolerance in the TG rats and AUC was very similar to CD group values (Table 1). Fasting insulin levels were significantly higher for both WT and TG rats from HCD groups (Fig. 2f). Two-way ANOVA analysis revealed a significant effect of the diet (*F*_(1,41)_ = 21.20; *p* < 0.0001) in both genotypes. HOMA-IR index was significantly greater for both WT and TG rats from hypercaloric diet groups compared to the control groups (Table 1). Nevertheless, these data suggest that HCD did not lead to the development of frank diabetes, yet led to the manifestation of a pre-diabetic state. In contrast, the HCD had a robust effect on lipid metabolism.

### Blood pressure

Systolic and diastolic blood pressure values obtained at 6 and 10 weeks of diet were not different between the experimental groups, indicating that no animal group showed signs of hypertension due to dietary intervention or genotype (Additional file 1: Figure S1).

### Behavioral assessment

We monitored cognitive performance using a spatial navigation version of MWM task. First testing was done prior to the assignment of different dietary regimens to assess baseline learning abilities of rats. The testing at the end of the study evaluated effects of the HCD-induced metabolic dysregulation alone and in combination with AD predisposing conditions on learning and memory.

### MWM and spatial learning preceding diet

At the end of the initial training period, 1 week prior to the start of the diet, all groups had learned the location of the platform to the same extent (Additional file 1: Figure S2A). Distance traveled in the target quadrant during the probe trial 1 following the learning was indicative of a good memory of the platform location (Additional file 1: Figure S2B).

### MWM and spatial relearning after diet

**Fig. 3:**
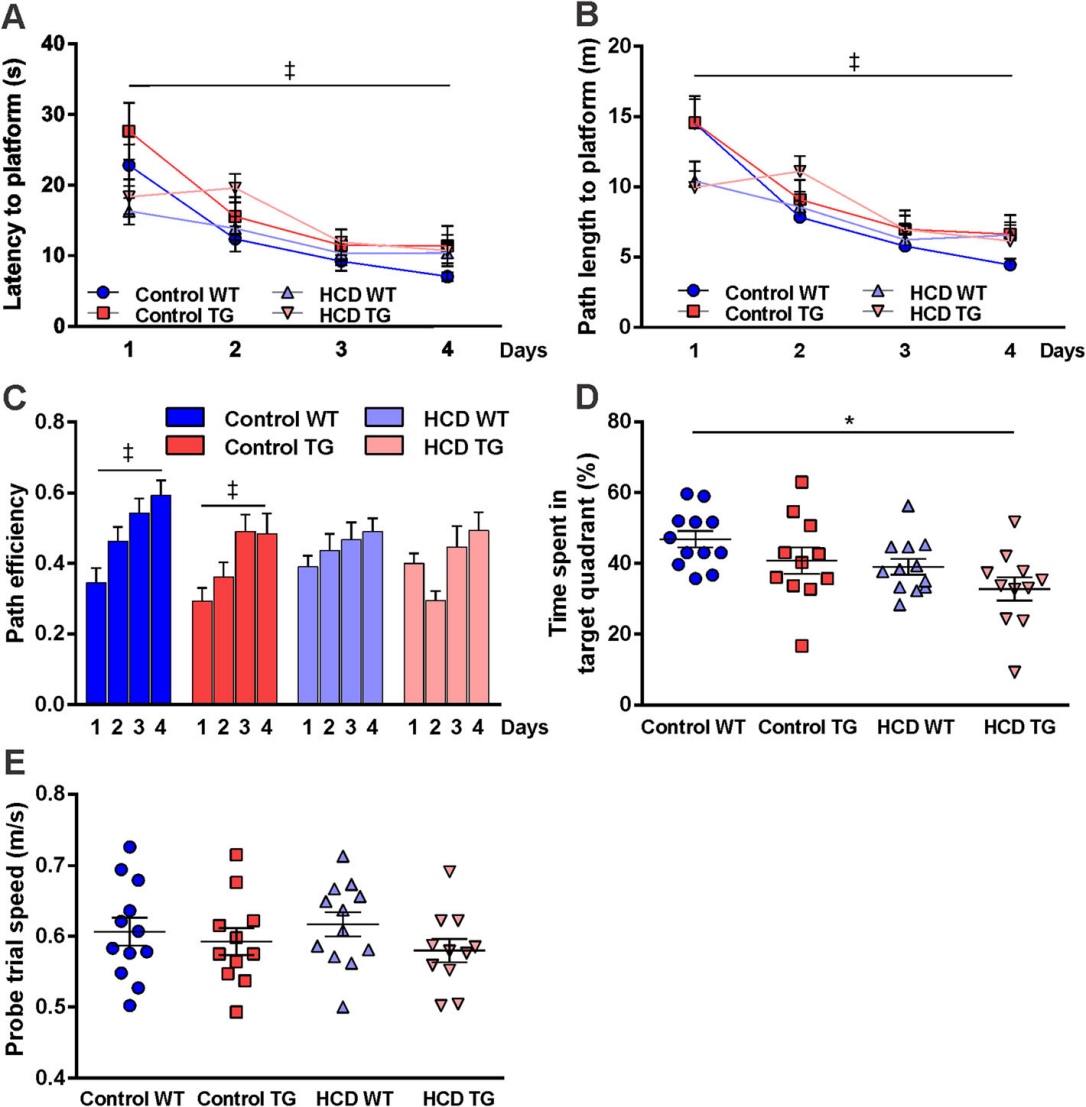
Morris water maze relearning and probe trial for memory test after 12 weeks on the diet. **a** Latency to platform in the 4-day training phase. **b** Mean path length to reach the platform in the 4-day training phase. **c** Path efficiency to reach the platform during 4 days of training. **d** Time spent in the target quadrant during the probe trial (Pr3) following relearning expressed as percent of total distance in probe trial. **e** Swim speed in the Pr3. Animal numbers are as follows: control WT (*n* = 12), control TG (*n* = 11), HCD WT (*n* = 12), HCD TG (*n* = 11). Values are presented as mean ± SEM. Significance is indicated by ‡ between days 1 and 4 in control groups, by * between HCD TG and control WT. RM two-way ANOVA, one-way ANOVA, Tukey’s multiple comparisons test, *p* < 0.05. *HCD* hypercaloric diet, *TG* transgenic, *WT* wildtype

Following 12 weeks on the diet, the latency to platform, path length to platform, and path efficiency were significantly improved in CD groups, but were not significantly improved in the HCD groups (Fig. 3a–c). TG rats on the HCD showed an inconsistent learning pattern with a sudden drop in path efficiency and increase in latency and distance on the second day of the task. However, by the end of the spatial acquisition phase, all rats learned the task to the same extent as indicated by the absence of differences between groups in any of these measurements at day 4 of training. Learning swim speed was comparable between the groups across days. During the probe trial (Probe 3), comorbid rats spent less time searching in the target quadrant, while the other groups had a preference for the quadrant where the platform was located during learning days. Tukey’s multiple comparisons test showed a significant decrease (*p* < 0.01; one-way ANOVA; Fig. 3d) in time spent in the target quadrant for the comorbid rats compared to control WT group. Swim speed did not differ between groups (Fig. 3e). Two-way ANOVA analysis revealed a significant effect of dietary treatment (*F*_(1,42)_ = 7.384; *p* < 0.01) and genotype (*F*_(1,42)_ = 4.462; *p* < 0.05) for time travelled in the target quadrant with no significant interaction, but the HCD TG group was significantly different from control WT group (*p* = 0.0085). Altogether, these results demonstrate diet- and genotype-dependent impairment in memory consolidation with a negative outcome in the comorbid condition.

### Open field test

Assessment of the effects of HCD alone and in conjunction with AD pathology on locomotion and anxiety level was done in the open field maze. Analysis of total ambulatory distance during a 20-min task did not result in any significant changes in the locomotor activity between groups (Additional file 1: Figure S3A); however, there was a genotype-dependent decrease (*F*_(1,43)_ = 6.371; *p* = 0.0154) in locomotor activity of TG rats. Time spent in the central zone of the open field arena as a measure of anxiety-like behavior was not affected by the diet. In contrast, the transgene significantly decreased (*F*_(1,42)_ = 10.09; *p* < 0.01) time spent in the central zone (Additional file 1: Figure S3B), suggesting that TG rats were more anxious.

### Neuroinflammation

Neuroinflammation is one of the earliest and most critical events occurring in the brain in response to insult and plays an important role in pathogenesis of AD. Microglia are the key cellular component of the inflammatory processes occurring in the brain and are the first ones to become activated and proliferate in response to disturbances in cerebral homeostasis. Astrocytes play a major role in maintaining brain health and get readily involved in inflammatory reactions. These two types of glial cells were included in our analysis as the elements of particular interest and were visualized using immunohistochemistry technique.

### Microglia activation

**Fig. 4:**
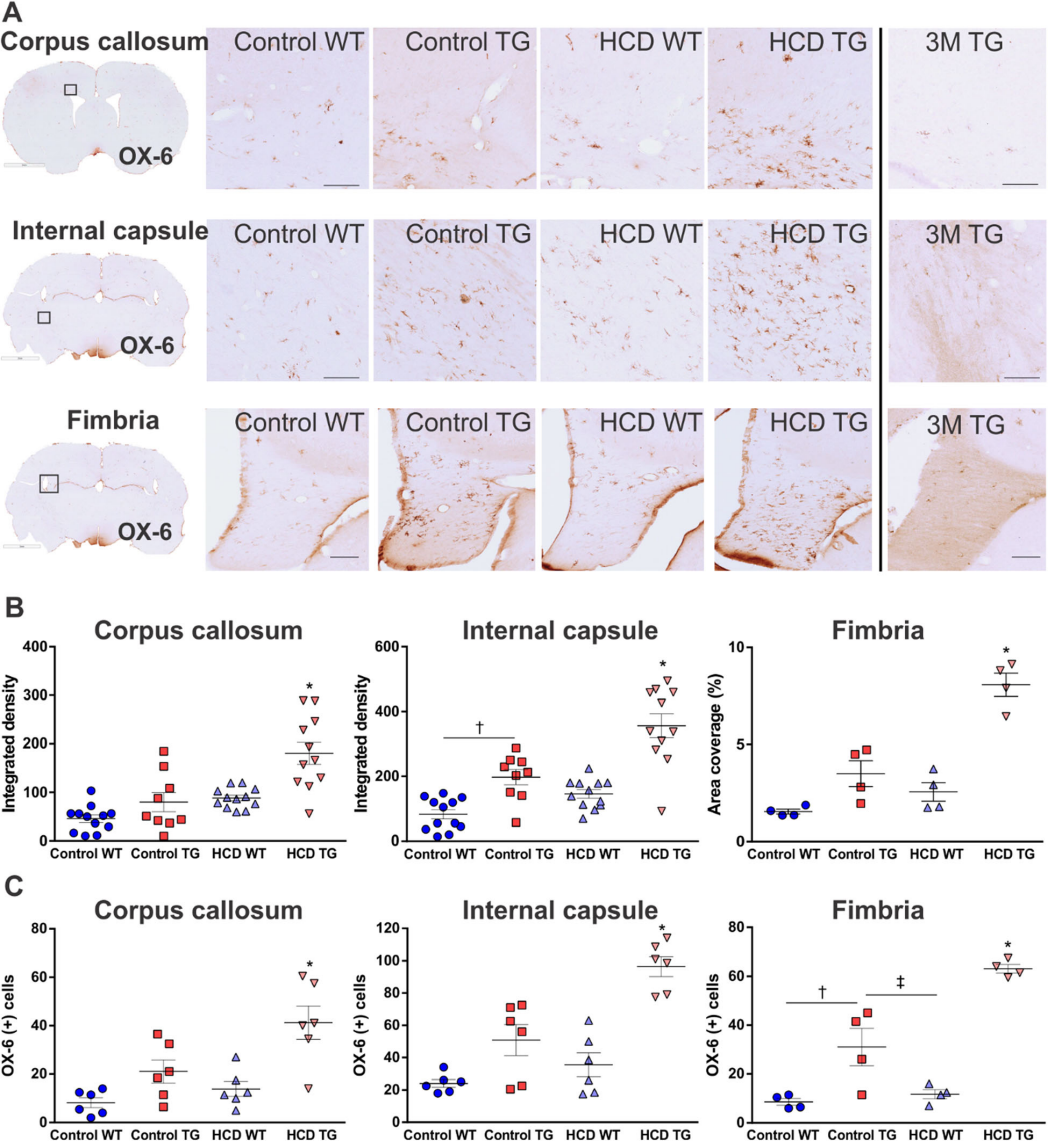
Activated microglia in white matter. **a** 10× photomicrographs of representative OX-6 immunolabelled activated microglial cells in the corpus callosum, internal capsule and fimbria hippocampi from the boxed regions indicated on the whole brain section insertion, right hemisphere. Photographs of the activated microglia in the three white matter regions of the three-month-old TG rat are shown in the right column. Scale bar 200μm. **b** Integrated density as a measure of microgliosis for corpus callosum and internal capsule. Animal numbers are as follows: control WT (*n* = 12), control TG (*n* = 11), HCD WT (*n* = 12), HCD TG (*n* = 11). Area coverage by a positive signal (as a percentage of a total area of the region) as a measure of microgliosis for fimbria. Animal numbers are *n* = 4 in each group. **c** OX-6 positive activated microglia cell counts in a field of area 100 mm^2^ in the in the corpus callosum, internal capsule and fimbria hippocampi. Values are presented as mean ± SEM. Significance is indicated by * between HCD TG and all other groups; by † between control groups; by ‡ between control TG and HCD WT groups. One-way ANOVA and Tukey’s multiple comparisons test, *p* < 0.05. *3M* three-month-old TG rat, *HCD* hypercaloric diet, *TG* transgenic, *WT* wildtype

**Fig. 5:**
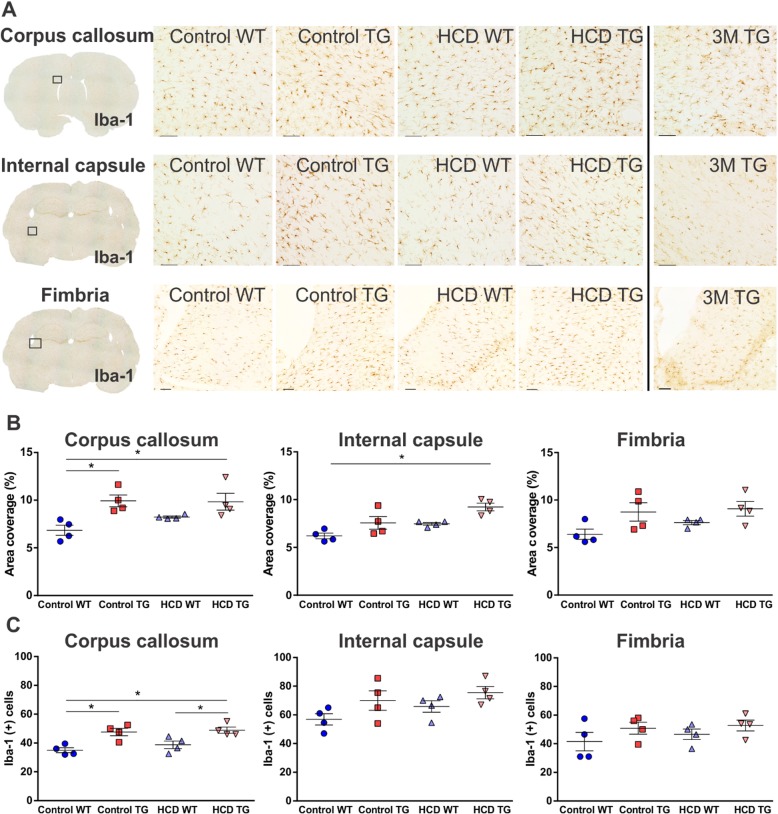
Total microglia in white matter. **a** 10× photomicrographs of representative Iba-1 immunolabelled microglial cells in the corpus callosum, internal capsule and fimbria hippocampi from the boxed regions indicated on the whole brain section insertion, right hemisphere. Photographs of the microglia in the three white matter regions of the 3-month-old TG rat are shown in the right column. Scale bar 100 μm. **b** Area coverage by a positive signal (as percentage of a total area of a region) for corpus callosum, internal capsule and fimbria. **c** Iba-1 positive microglia cell counts in a field of area 100 mm^2^ in the in the corpus callosum, internal capsule, and fimbria hippocampi. Animal numbers are as follows: control WT (*n* = 4), control TG (*n* = 4), HCD WT (*n* = 4), HCD TG (*n* = 4). Values are presented as mean ± SEM. Significance is indicated by * for control WT vs. both TG groups in the corpus callosum (in **b**, **c**), HCD TG vs HCD WT in corpus callosum (in **c**), and control WT vs HCD TG in the internal capsule (in **b**). One-way ANOVA and Tukey’s multiple comparisons test, *p* < 0.05. *3M* three-month-old TG rat, *HCD* hypercaloric diet, *TG* transgenic, *WT* wildtype

We looked for signs of microglial inflammation by scanning the entire brain from all frontal to posterior levels. The pathology observed was located mainly in the white matter regions with very few activated microglia cells observed in the gray matter regions such as the cortex and hippocampus. There were no apparent differences among the groups. Microglia activation in the white matter, detected with the OX-6 immunostaining, has been shown to undergo an age-related increase in the TG rats compared to WT rats [31]. The images of the OX-6 activated microglia in three white matter regions from the 3-month-old TG animal demonstrate that there is a low activation of microglia in the young animal (Fig. 4a), similar to that of the WT aged rat. These images were complemented with an Iba-1-positive microglia cells from the young TG animal (Fig. 5a).

A detailed immunohistochemical assessment of the brain sections indicated significant changes in white matter inflammation due to the combination of the diet and transgene. The comorbid condition of HCD in the TG group resulted in a large increase in OX-6 positive activated ramified microglia in all subcortical white matter areas examined, including corpus callosum (starting as far anterior as the forceps minor), internal capsule, anterior commissure, optic tract, and fimbria of the hippocampi. Representative images are shown in Fig. 4a. HCD TG group had significant microgliosis in all white matter regions compared to all other groups (Fig. 4b, c). For the HCD TG compared to the control WT group, the *p* value was less than 0.0001 for all regions. Within the TG groups, the HCD TG was significant compared to the control TG groups with *p =* 0.0003 (*p =* 0.0259 for cell counts) in the corpus callosum and *p =* 0.0002 (*p ≤* 0.0008 for cell counts) in the internal capsule and fimbria.

This white matter microglial activation was also genotype-dependent, with APP21TG rats showing significantly higher OX-6-positive signal (integrated density/area coverage and cell number) in comparison to WT rats in the corpus callosum (*F*_1,40_ = 17.84, *F*_1,20_ = 19.23 for cell count; *p* ≤ 0.0003 both), internal capsule (*F*_1,40_ = 49.03, *F*_1,20_ = 40.02 for cell count; *p* < 0.0001 both), and fimbria (*F*_1,12_ = 53.17, *F*_1,12_ = 80.42 for cell count; *p* < 0.0001 both). In addition, two-way ANOVA analysis showed a significant effect of diet on microgliosis in the corpus callosum (*F*_1,40_ = 22.88, *p* < 0.0001; *F*_1,20_ = 7.96, *p =* 0.01 for cell count), internal capsule (*F*_1,40_ = 22.89, *F*_1,20_ = 17 for cell count; *p* < 0.0001), and fimbria (*F*_1,12_ = 29.73; *F*_1,20_ = 17 for cell count; *p* ≤ 0.0001 both). There was also a significant diet-genotype interaction on microgliosis in the internal capsule (*F*_1,40_ = 4.250, *p* = 0.0458 and *F*_1,20_ = 6.06, *p =* 0.023 for cell count) and fimbria (*F*_1,12_ = 12.09, *p =* 0.0046 and *F*_1,12_ = 12.43, *p =* 0.0042 for cell count), and almost significant interaction in the corpus callosum (*F*_1,40_ = 3.809, *p =* 0.0588).

Comorbid impact on total microglia within the white matter tracts was also assessed (Fig. 5). The comorbid HCD TG group had significantly greater area coverage by Iba-1-positive signal and more microglia cells than the Control WT group for both the corpus callosum (*p* < 0.02 for both) and the internal capsule (area coverage *p =* 0.0013; cell count *p =* 0.08), changes not seen in the fimbria (Fig. 5b, c). In the corpus callosum, control TG rats also had greater numbers of microglia than control WT animals (*p* < 0.016 for both).

Iba-1 stained section analysis indicated a significant transgene-dependent increase in area coverage by Iba-1-positive microglia in the corpus callosum (*F*_1,12_ = 15.13; *p =* 0.0021), internal capsule (*F*_1,12_ = 13.73; *p =* 0.003), and fimbria (*F*_1,12_ = 7.684; *p =* 0.0169; Fig. 5b). There was an additional effect of the diet on the microgliosis in the internal capsule (*F*_1,12_ = 12.04; *p =* 0.0046). The genotype also had a similar effect on the number of microglia in the corpus callosum (*F*_1,12_ = 24.3; *p =* 0.0003) and internal capsule (*F*_1,12_ = 5.4; *p =* 0.038), but not in the fimbria (Fig. 5c).

Thus, comorbid rats demonstrated a large microglial activation in all white matter areas analyzed along with an increase in microglial proliferation in the corpus callosum and internal capsule. Additional analysis revealed transgene-related effects on microglial activation and proliferation in all white matter regions. There was a diet-induced activation in all regions and proliferation in the internal capsule.

### Astrogliosis

**Fig. 6:**
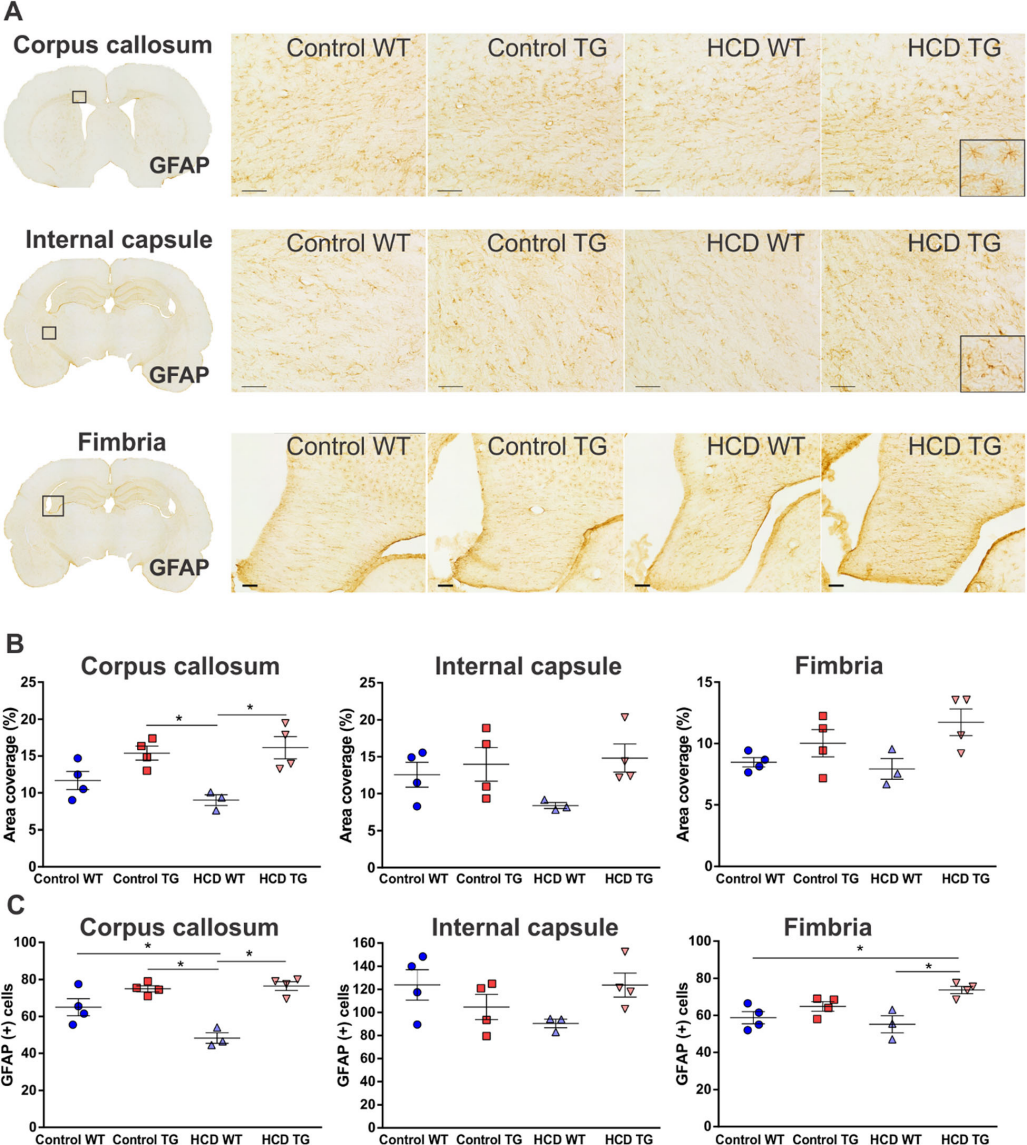
Reactive astrocytosis in white matter. **a** 10× photomicrographs of representative GFAP immunolabelled astrocytes in the corpus callosum, internal capsule and fimbria hippocampi. Scale bar 100 μm. Magnified images of individual astrocytes are inserted at the bottom right corner of image panels in **a**. **b** Area coverage by a positive signal (as percentage of a total area of a region) for corpus callosum, internal capsule and fimbria. Animal numbers are as follows: control WT (*n* = 4), control TG (*n* = 4), HCD WT (*n* = 3), HCD TG (*n* = 4). Values are presented as mean ± SEM. Significance is indicated by * for HCD WT vs both TG groups (in **b**) and additionally vs. control WT in the corpus callosum (in **c**); HCD TG vs both WT groups in the internal capsule (in **c**). One-way ANOVA and Tukey’s multiple comparisons test, *p* < 0.05. *HCD* hypercaloric diet, *TG* transgenic, *WT* wildtype

Area of coverage by GFAP-immunopositive astroglia expressed as a percentage of total area of ROI was taken as a measurement of astrocyte reactivity in subcortical white matter (Fig. 6a, b). For the comorbid HCD TG group, the only observed increase in astrocyte reactivity was compared to the HCD WT group in the corpus callosum (*p =* 0.0095). In the corpus callosum, even the control TG group had higher levels of astrocyte reactivity compared to the HCD WT (*p =* 0.0188). There was also a significant increase in astrocyte number in the corpus callosum in HCD TG (*p =* 0.0004), control TG (*p =* 0.0007), and control WT (*p =* 0.0195) groups compared to HCD WT rats (Fig. 6c). Additionally, there was a greater number of cells in the fimbria of comorbid rats when compared to both WT groups (control *p =* 0.0181, HCD (*p =* 0.0075).

There was a transgene effect in that TG rats showed a significant increase in both astrocyte reactivity (*F*_1, 11_ *=* 20.05, *p =* 0.0009) and density (*F*_1, 11_ *=* 36.06, *p* < 0.0001) in the corpus callosum and fimbria hippocampi (*F*_1, 11_ = 8.307, *p =* 0.0149 for reactivity; *F*_1, 11_ = 16.3.8, *p =* 0.0019 for density), compared to WT groups.

### Other markers of neuroinflammation

To further analyze neuroinflammation, we performed immunohistochemical staining for iNOS, a pro-inflammatory marker of various cells including glia induced by stimuli such as cytokines, and CD 206, an anti-inflammatory biomarker of macrophage/microglia cells involved in phagocytosis and inflammatory response resolution (Additional file 1: Figure S4). Microscopic analysis of the staining revealed no differences in the expression of both markers between the experimental groups. In fact, there were only a few iNOS-positive cells observed in the brain tissue (Additional file 1: Figure S4A, magnified image insertion), and no positive white matter cellular signal was detected in the CD 206 staining (Additional file 1: Figure S4B).

### Neuronal density

**Fig. 7:**
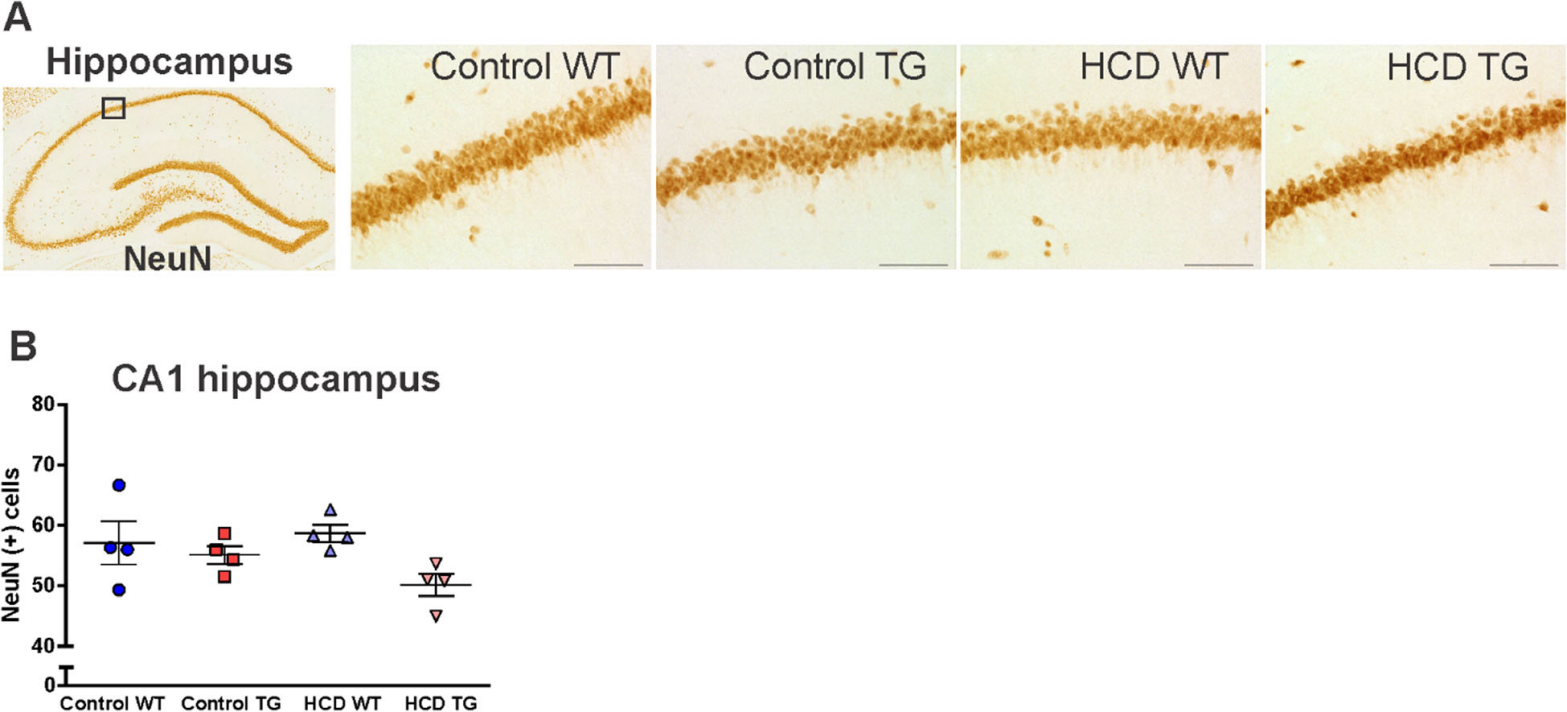
Neuronal counts identified by neuronal nuclear antigen (NeuN) immunohistochemistry*.* **a** 20× photomicrographs of the dorsal hippocampus CA1 subregion pyramidal neurons. Scale bar 100 μm. Boxed area corresponds to a field defined for cell counts. **b** NeuN-positive cell counts in a field of area 0.2 mm^2^. Animal numbers are *n* = 4 in each group. Values are presented as mean ± SEM. *HCD* hypercaloric diet, *TG* transgenic, *WT* wildtype. One-way ANOVA, Tukey’s multiple comparisons test, *p* < 0.05

Dorsal hippocampus, particularly the CA1 region, is a crucial structure for spatial learning and memory and is very susceptible to the pathological processes in AD [37–40]. We assessed whether there is a loss of neurons in the CA1 subregion of the hippocampus (Fig. 7a). Counts of NeuN-positive pyramidal neurons revealed no differences in the neuronal density between experimental groups (Fig. 7b).

### Synaptic density

**Fig. 8:**
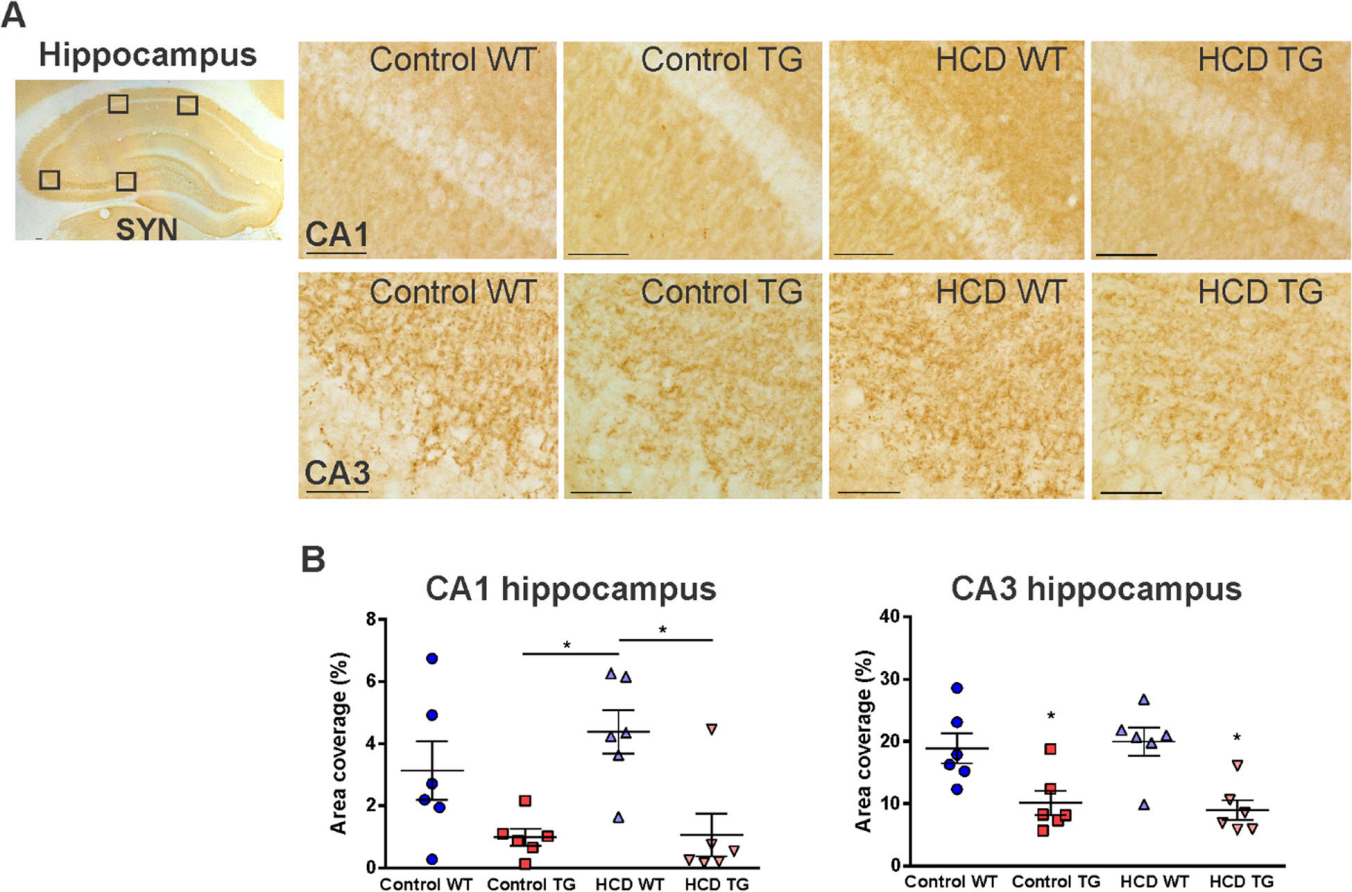
Synaptic density in the hippocampus identified by synaptophysin (SYN) immunohistochemistry. **a** 20× photomicrographs of the pyramidal neurons in the dorsal hippocampus CA1 (top row) and CA3 (bottom row) subregions. Scale bar 50 μm. Boxed area corresponds to a field defined for quantification. **b** Synaptophysin area coverage (%) in CA1 and CA3 regions of the hippocampus. Animal numbers are *n* = 6 in each group. Values are presented as mean ± SEM. Significance is indicated by * for HCD WT vs both TG groups in CA1 and for both TG groups vs both WT groups in CA3 region. *HCD* hypercaloric diet, *TG* transgenic, *WT* wildtype. One-way ANOVA, Tukey’s multiple comparisons test, *p* < 0.05

Synaptic density was analyzed in the CA1 and CA3 dorsal hippocampal subregions using synaptophysin immunostaining to detect synaptic vesicles (Fig. 8a). The area of coverage by a positive signal was significantly decreased in the TG rats compared to the WT animals in both regions (CA1 *p =* 0.0008, *F*_(1,20)_ *=* 15.38; CA3 *p =* 0.0001, *F*_(1,20)_ *=* 22.60; Fig. 8b). In the TG rats that were also on the HCD, there was no additional effect of the co-morbidity on the synaptic density in any of the regions. Rats from the HCD TG and control TG groups showed significantly lower synaptic density compared to the HCD WT (*p =* 0.0126 and 0.0148, respectively) in the CA1 region, and to the HCD WT (*p =* 0.0153 and 0.0065, respectively) and control WT (*p =* 0.0342 and 0.0149, respectively) in the CA3 region.

### Myelination

Activated microglia were highly accumulated in the cerebral white matter of TG rats on HCD with some more minor transgene and diet effects. To assess if signs of demyelination of the white matter tracts were present at this level glial pathology, Luxol fast blue staining was performed (Additional file 1: Figure S5A, B). We quantified the percentage of area coverage by a positive signal for both corpus callosum (Additional file 1: Figure S5C) and internal capsule (Additional file 1: Figure S5D). There was no statistically significant difference in myelin content between the groups and no effect of genotype or diet was detected. Thus, increased microglial activation was not accompanied by loss of myelin at this stage.

### Cerebral amyloid-β deposition

Analysis of immunohistochemically stained sections revealed that no amyloid-β fibrillar accumulates in the brain tissue in the experimental groups including comorbid rats, suggesting that the HCD in the TG did not lead to detectable human amyloid aggregation and deposition in the brain tissue (Additional file 1: Figure S6).

## Discussion

The results of this investigation clearly show, for the first time, that APP21 TG predisposed to AD rats maintained on a high-fat and high-carbohydrate diet not only develop considerable metabolic perturbations, but they also exhibit marked widespread white matter microgliosis that was accompanied by impairment on a spatial memory task compared to the performance level of wildtype rats. However, there was no neuronal loss or further decrease in synaptic density in the hippocampus of these comorbid rats. Although there were some behavioral, synaptic, and inflammatory changes that could be attributed to the diet or the transgene alone, it was clear that the more significant neuroinflammation and memory and learning deficits were due to the combination of the energy-rich high-fat, high-carbohydrate diet, and the TG condition. This is the first demonstration of the impact of hypercaloric diet on white matter in a vulnerable aging brain with increased levels of pathogenic hAPP. These TG rats have been previously characterized to have dense neuronal staining for hAPP, but no evidence of plaques [30, 33]. Amyloid-β plaque deposits were also not detected in the TG and comorbid rats in this study. This differs from previous mouse models that assessed high-fat die-induced metabolic syndrome on animals with established classical AD events including amyloid plaque and tau pathology.

The hypercaloric diet approach was chosen to mimic a modern dietary pattern in the human population represented by a combination of food that is high in fat and simple sugars and carbohydrate-rich beverages [10, 41]. This study was not designed to examine the exact effects of the specific source of fat or type of fatty acids or specific carbohydrates ingested in a large amount. The intent was to examine a combined diet with a high content of both components to deliver an excess of calories associated with induction of metabolic syndrome pathology in our rat model [10, 41, 42]. We therefore cannot extrapolate on the potential effects of high-fat diet or high-carbohydrate diet in isolation.

Twelve weeks on the HCD were sufficient for the development of significant obesity and visceral adiposity in these rats. While rats in control groups had normal rat chow as the only source of energy, rats maintained on high-fat, high-sugar diet had an additional energy uptake from a corn syrup drink, which resulted in a reduction of food consumption in these animals, but nonetheless a greater total caloric intake per rat compared to rats on CD.

The ingestion of high-fat and high-carbohydrate calories had effects in the periphery and markedly altered lipid metabolism, increasing triglycerides, total cholesterol, and atherogenic non-HDL fraction in rats of both genotypes. Rats TG for hAPP were more susceptible to these changes and had a greater degree of dyslipidemia. In contrast, WT rats were more prone to perturbations in glucose metabolism. Such sensitivity of lipid homeostasis to a long-term consumption of high-fat diets has been shown previously [41] and has also been reported for the Fischer 344 rat strain [23, 43]. However, this is the first instance where the high-fat high-sugar diet has been tested in the APP21 TG rat demonstrating a greater degree of dyslipidemia compared to the WT subjects.

Although fasting glucose levels were within a normal range for both groups on HCD, the HCD led to the increase of fasting insulin levels suggesting the development of hyperinsulinemia and insulin resistance in rats of both genotypes.

During a 2-h glucose tolerance test, WT rats had sustained high blood glucose levels indicating a decreased tolerance for glucose in this group. Interestingly, HCD did not appear to induce pronounced glucose intolerance in TG rats, at least not after the 12-week-long intervention. This physiological difference in response to excessive caloric intake could implicate mutated hAPP gene inserted in the genome of rats and overexpressed in tissues other than brain (i.e., liver, kidney, lung) and its possible interaction with mechanisms of metabolism. Similar to our observation, 5xFAD mice bearing five human familial AD mutations including APP_Swe_ placed on a high-fat diet for 10 weeks did not show signs of glucose intolerance in the oral version of the test compared to control WT group [44]. The presence of carbohydrate metabolism alterations has been reported in patients with symptomatic AD, in which there are lower rates of fasting blood glucose as well as lower glucose values in the oral glucose tolerance test [45]. However, this unique phenomenon would need further separate investigation using more sensitive methods to find out whether there is a difference in glucose metabolism and in the role of compensatory mechanisms to overcome dietary effects between the two genotypes, which could account for this diverse response to a glucose load and was not in the focus of the present study.

There was no dietary effect on the blood pressure, demonstrating that a 12-week exposure to the HCD was not long enough to develop hypertension in this rat strain. However, the non-invasive method to measure blood pressure used in this study falls short of the accuracy of invasive techniques, and might be insensitive to subtle early changes in blood pressure possibly present at this stage.

Behavioral analysis at the end of the diet indicated an impact of the diet-induced metabolic alterations on memory consolidation in rats with AD predisposition, however only compared the control WT rats. This observation clearly has implications for human populations with a high prevalence of obesity due to a hypercaloric Western style diet with advancing age and increasing levels of brain amyloid [18, 46]. Studies using TG AD mouse models have shown similar effects of high-fat diet on the spatial memory domain and noted the link of these effects to the inflammatory events [44, 47–49]. One study showed increased microglia activation detected in vivo using positron emission tomography, and a greater amyloid plaque load in APP/PS1 TG mouse which received a high-fat diet and a streptozocin treatment [49]. Interestingly, a triple-transgenic AD mouse just on a high-fat diet did not exhibit increase in amyloid plaque deposition or tau-pathology, rather a significantly increased number of activated microglia associated with plaques in the hippocampal region that was suggested to be the primary mediating pathology to an observed cognitive impairment [47]. Another study using a APPswe/PS1 TG mouse of AD similarly showed no effect of the Western diet on the brain parenchymal amyloid burden; however, the diet resulted in decreased synaptic plasticity and blood–brain barrier dysfunction which could contribute to the behavioral deficits [48]. These changes were attributed to the systemic inflammation promoted by the Western diet [48]. This is in line with studies of human brain showing a lack of correlation between amyloid plaque burden and presence or severity of dementia symptoms [50, 51]. This suggests that other events contribute to manifestation and progression of cognitive decline and that neuroinflammation including white matter microgliosis and astrogliosis can be among them [52].

As the field of AD research has started to move away from the amyloid causal hypothesis, the white matter inflammation and other white matter changes concepts have been gaining attention and recognition as important players in cognitive impairment [3, 7, 53, 54]. White matter abnormalities visualized as hyperintensities on MRI scans are common findings among the elderly population. These signals increase with aging; are often present in mild cognitive impairment (MCI), AD, and patients with metabolic disorders; and have been shown to highly correlate with cognitive decline [8, 55, 56]. Of great interest is the clinical finding that white matter lesions tend to be present well before symptoms of cognitive deterioration start to appear [7, 8, 54]. This has opened a new avenue to explore the potential of cerebral white matter lesions as a new biomarker of cognitive impairment such as MCI and AD dementia and a possible target for prevention and therapy.

Our results clearly indicate an increased microgliosis and microglial proliferation in the white matter tracts of TG rats expressing pathogenic hAPP markedly aggravated by diet-induced metabolic dysregulations in the comorbid rats. Analysis of the brain tissue has shown a widespread inflammation of the white matter, including the corpus callosum, fimbria, internal capsule, cingulum, anterior commissure, and optic tract. This finding is of considerable interest as it replicates the white matter pathology associated with advanced age, MCI, early AD, and metabolic disorders in the human population [3, 5, 6]. Intriguingly, the white matter inflammation appeared to be an early pathological event as there was no apparent loss of CA1 hippocampal neurons or decrease in synapses in the CA1 and CA3 subregions of the hippocampus in the comorbid animals at this stage of the disease.

Additional analysis of pro-inflammatory and anti-inflammatory glia markers iNOS and CD 206, respectively, indicated that there was no increase in expression of these markers in the TG condition alone or in the comorbidity with the HCD. Both markers have been shown to be upregulated in response to ischemic insult and traumatic brain injury, conditions associated with neuroinflammatory process [57, 58]. Temporally, after the initial upregulation of expression of both biomarkers shortly after an insult, there is a gradual decrease of anti-inflammatory phenotype of responding cells (primarily glia) with maintained increased expression of the pro-inflammatory profile over a couple of weeks. In contrast, inflammatory responses to acute systemic infection, sepsis, is characterized by a great increase in iNOS and no change in CD 206 expression [59, 60]. Neurodegenerative diseases, including AD, have been associated with mixed activation glia phenotype and rather minimal change to the iNOS expression [61]. This suggests that the inflammatory marker expression profile may differ depending not only on the timepoint analyzed/disease stage but also on the type of injury, which might involve different pathways in the pathogenesis. Aging and related senescence of the immune system including glia likely further contributes to these differences. The iNOS and CD206 marker expression profile of glial cells observed in our model after 12 weeks on the diet could represent a specific phenotype (increased pro-inflammatory marker OX-6) associated with a chronic low-grade systemic inflammation. It is possible that an acute response to the HCD might bear a different profile including a transient initial change of the particular markers.

In the present study, we also assessed myelination of two major white matter tracts, the corpus callosum and internal capsule, which appeared to be unchanged in APP21 TG rats on the HCD. Further analysis confirmed that the white matter microgliosis was not accompanied by signs of myelin loss at this stage. Nevertheless, axonal damage or perturbation to oligodendrocyte health could begin to develop and should be examined in the future studies to enhance understanding of the white matter pathological changes.

Additional brain tissue analysis should be carried out in order to identify the nature and magnitude of the inflammatory events as well as determine if these inflammatory events are precursors to or consequences of potential vascular changes and other processes that might take place at this early stage of dietary intervention and contribute to the cognitive dysfunction. However, these elements of interest were not in the focus of the present study which aimed to address the effects of HCD superimposed on the high amyloid background on the major glial cells, microglia and astrocyte, activation as an indicator of neuroinflammatory process.

Clinical data points toward an association of cerebral white matter pathology with perturbations in executive function, processing speed, and general cognition [62]. Widespread neuroinflammatory responses to the HCD, primarily denoted by microgliosis and increased microglia cell activation, seen in the white matter of TG rats may interfere with functioning of multiple cognitive domains leading to a general decline and may contribute to the observed impairment in the behavioral task performance. However, to establish a clear connection between the white matter inflammation and cognitive impairment, more studies including neuronal health assessment should be performed. The spatial navigation version of the MWM used in the present study was chosen to assess learning and memory dependent on hippocampal formation that is highly vulnerable to AD pathology. However, it is not the most sensitive for specific testing of executive function components that might be affected at the prodromal stage of the disease in our TG rat model. It will be necessary to perform more sensitive tests (e.g., operant conditioning based set-shifting task) to clarify the cognitive deficits that may be related to the observed brain white matter pathology.

The sex-dependent differences in the effect of metabolic syndrome on neuroinflammation and other early pathology of pre-AD and cognition were not tested in the current study using only male rats. Future projects should consider including experiments conducted on female animals to address the potential role of biological sex and endocrinological differences in the interaction of these conditions.

## Conclusions

Our study using a TG APP21 rat on HCD suggests the role of diet-induced metabolic alterations as a risk factor for white matter inflammation, which is an early brain pathology in MCI and AD, as a possible point of interaction with prodromal phase AD. Results further suggest that white matter inflammation may lead to accelerated development of cognitive symptoms, since the white matter microglial activation was accompanied by cognitive impairment in comorbidity condition compared to normal rats from WT population. The other two groups, TG rats on the CD and WT on the HCD, did not demonstrate this significant cognitive change from the WT CD animals. Activated inflammatory cells were mainly located in the white matter which raises a number of important questions on the nature of events and mechanisms that trigger this specific response. The intense white matter inflammatory response provoked by the dietary intervention in the TG rats also suggests that specific anti-inflammatory agents may be a potential treatment and preventative strategy. Several approaches could be taken in this therapeutic direction including targeting inflammatory cytokines or components of the arachidonic acid pathway that mediate the inflammation.

## Supplementary information

**Additional file 1: Figure S1**. *Arterial blood pressure measured three weeks prior to the diet onset, 6 and 10 weeks on the diet.* A) Systolic and B) diastolic blood pressure levels. **Figure S2**. *Morris water maze learning and memory test performed one week prior to the diet onset.* A) Latency to reach the platform in the 4-day training phase. B) Time spent in the target quadrant during the probe trial following the learning phase (Pr1) expressed as percent of total time in probe trial. **Figure S3**. *Locomotor activity and anxiety-like behavior in open filed test.* (A) Total ambulatory distance for 20 minutes and (B) percentage of time spent in central zone of an open field arena. **Figure S4**. *Pro-inflammatory and anti-inflammatory markers in white matter.* 10× photomicrographs of representative iNOS (A) and CD 206 (B) immunolabelled microglial cells in the corpus callosum and internal capsule - boxed regions on coronal brain sections, right hemisphere. Scale bar 100μm. Magnified image of individual iNOS positive cell is inserted on image of Control WT rat in panel A. Images of a positive control for iNOS and CD 206 staining showing positively stained cells in post-stroke striatum is included in respective panels. **Figure S5**. *Myelination of white matter.* 10× photomicrographs of representative brain sections stained with Luxol fast blue containing (A) corpus callosum and (B) internal capsule, right hemisphere. Scale bar 100μm. Area coverage by a positive signal (as percentage of a total area of a region) for (C) corpus callosum and (D) internal capsule. **Figure S6**. Immunohistochemical staining for cerebral amyloid-β deposition. 10× photomicrographs of representative brain sections stained with 4G8 containing hippocampus (CA1 region), cerebral cortex and corpus callosum (periventricular region), right hemisphere. A human tissue sample from a patient with confirmed Alzheimer's disease used as a positive control. Scale bar 100 μm.

## Data Availability

The datasets supporting the conclusions of this article are included within the article and its additional files.

## Abbreviations

AD: Alzheimer’s disease
APP: Amyloid precursor protein
AUC: Area under the curve
Aβ: Amyloid-β peptide
CD: Control diet
GFAP: Glial fibrillary acidic protein
HCD: High calorie diet
HDL: High-density lipoprotein cholesterol
IGTT: Intraperitoneal glucose tolerance test
MCI: Mild cognitive impairment
MHC: Major histocompatibility complex
MWM: Morris water maze
TG: Transgenic
WT: Wildtype

## References

[1] Querfurth HW, LaFerla FM (2010). Mechanisms of disease. Alzheimer’s disease. N Engl J Med.

[2] Heppner FL, Ransohoff RM, Becher B (2015). Immune attack: the role of inflammation in Alzheimer disease. Nat Rev Neurosci.

[3] Raj D, Yin Z, Breur M, Doorduin J, Holtman IR, Olah M (2017). Increased white matter inflammation in aging- and Alzheimer’s disease brain. Front Mol Neurosci.

[4] Englund E, Brun A (1990). White matter changes in dementia of Alzheimer’s type: the difference in vulnerability between cell compartments. Histopathology..

[5] Simpson JE, Fernando MS, Clark L, Ince PG, Matthews F, Forster G (2007). White matter lesions in an unselected cohort of the elderly: astrocytic, microglial and oligodendrocyte precursor cell responses. Neuropathol Appl Neurobiol.

[6] Simpson JE, Ince PG, Higham CE, Gelsthorpe CH, Fernando MS, Matthews F (2007). Microglial activation in white matter lesions and nonlesional white matter of ageing brains. Neuropathol Appl Neurobiol.

[7] Lee S, Viqar F, Zimmerman ME, Narkhede A, Tosto G, Benzinger TLS (2016). White matter hyperintensities are a core feature of Alzheimer’s disease: evidence from the dominantly inherited alzheimer network. Ann Neurol.

[8] Prins ND, Scheltens P (2015). White matter hyperintensities, cognitive impairment and dementia: an update. Nat Rev Neurol.

[9] Cechetto DF, Hachinski VC, Whitehead SN (2008). Vascular risk factors and Alzheimer ’s disease. Expert Rev Neurother.

[10] Cordain L, Eaton SB, Sebastian A, Mann N, Lindeberg S, Watkins BA (2005). Origins and evolution of the Western diet : health implications for the 21st century. Am J Clin Nutr.

[11] Tappy L, Le K-A (2010). Metabolic effects of fructose and the worldwide increase in obesity. Physiol Rev.

[12] Saklayen MG (2018). The global epidemic of the metabolic syndrome. Curr Hypertens Rep.

[13] Kivipelto M, Ngandu T, Fratiglioni L, Viitanen M, Kåreholt I, Winblad B (2005). Obesity and vascular risk factors at midlife and the risk of dementia and Alzheimer disease. Arch Neurol.

[14] Tsai C-K, Kao T-W, Lee J-T, Wu C-J, Hueng D-Y, Liang C-S (2016). Increased risk of cognitive impairment in patients with components of metabolic syndrome. Medicine (Baltimore).

[15] Vanhanen M, Koivisto K, Moilanen L, Helkala E-L, Hanninen T, Soininen H (2009). Association of metabolic syndrome with Alzheimer disease. Neurology.

[16] Razay G, Vreugdenhil A, Wilcock GK (2007). The metabolic syndrome and Alzheimer disease. Metab Syndr Obes.

[17] Tolppanen A-M, Solomon A, Soininen H, Kivipelto M (2012). Midlife vascular risk fctors and Alzheimer’s disease: evidence from epidemiological studies. J Alzheimers Dis.

[18] Whitmer RA, Gunderson EP, Barrett-Connor E, Quesenberry CP, Yaffe K (2005). Obesity in middle age and future risk of dementia: a 27 year longitudinal population based study. Br Med J.

[19] Panza F, Frisardi V, Seripa D, P. Imbimbo B, Sancarlo D, D’Onofrio G (2011). Metabolic syndrome, mild cognitive impairment and dementia. Curr Alzheimer Res.

[20] Frisardi V, Solfrizzi V, Seripa D, Capurso C, Santamato A, Sancarlo D (2010). Metabolic-cognitive syndrome: a cross-talk between metabolic syndrome and Alzheimer’s disease. Ageing Res Rev.

[21] Viticchi G, Falsetti L, Buratti L, Luzzi S, Bartolini M, Acciarri MC (2015). Metabolic syndrome and cerebrovascular impairment in Alzheimer’s disease. Int J Geriatr Psychiatry.

[22] Freeman LR, Hayley-Zitlin V, Granholm A-C (2011). Diet-induced effects on neuronal and glial elements in the middle-aged rat hippocampus. Nutr Neurosci.

[23] Granholm A-C, Bimonte-Nelson HA, Moore AB, Nelson ME, Freeman LR, Sambamurti K (2008). Effects of a saturated fat and high cholesterol diet on memory and hippocampal morphology in the middle-aged rat. J Alzheimers Dis.

[24] Kanoski SE, Zhang Y, Zheng W, Davidson TL (2010). The effects of a high-energy diet on hippocampal function and blood-brain barrier integrity in the rat. J Alzheimers Dis.

[25] Molteni R, Barnard RJ, Ying Z, Roberts CK, Gómez-Pinilla F (2002). A high-fat, refined sugar diet reduces hippocampal brain-derived neurotrophic factor, neuronal plasticity, and learning. Neuroscience..

[26] Soares E, Prediger RD, Nunes S, Castro AA, Viana SD, Lemos C (2013). Spatial memory impairments in a prediabetic rat model. Neuroscience..

[27] Bhat NR (2010). Linking cardiometabolic disorders to sporadic Alzheimer’s disease: A perspective on potential mechanisms and mediators. J Neurochem.

[28] Jayaraman A, Pike CJ (2014). Alzheimer’s disease and type 2 diabetes: multiple mechanisms contribute to interactions. Curr Diab Rep.

[29] Misiak B, Leszek J, Kiejna A (2012). Metabolic syndrome, mild cognitive impairment and Alzheimer’s disease-The emerging role of systemic low-grade inflammation and adiposity. Brain Res Bull.

[30] Agca C, Fritz JJ, Walker LC, Levey AI, Chan AW, Lah JJ (2008). Development of transgenic rats producing human β-amyloid precursor protein as a model for Alzheimer’s disease: transgene and endogenous APP genes are regulated tissue-specifically. BMC Neurosci.

[31] Weishaupt N, Liu Q, Shin S, Singh R, Agca Y, Agca C (2018). APP21 transgenic rats develop age-dependent cognitive impairment and microglia accumulation within white matter tracts. J Neuroinflammation.

[32] Rosen RF, Fritz JJ, Dooyema J, Clintron AF, Hamaguchi T, Lah JJ (2012). Exogenous seeding of cerebral β-amyloid in βAPP -transgenic rats. J Neurochem.

[33] Klakotskaia D, Agca C, Richardson RA, Stopa EG, Schachtman TR, Agca Y (2018). Memory deficiency, cerebral amyloid angiopathy, and amyloid-β plaques in APP+PS1 double transgenic rat model of Alzheimer’s disease. PLoS One.

[34] Antunes LC, Elkfury JL, Jornada MN, Foletto KC, Bertoluci MC (2016). Validation of HOMA-IR in a model of insulin-resistance induced by a high-fat diet in Wistar rats. Arch Endocrinol Metab.

[35] Watson C, Paxinos G. The rat brain in stereotaxic coordinates. 6th ed. Elsevier Inc. Burlington, MA, USA Academic press; 2007.

[36] Carriel V, Campos A, Alaminos M, Riamondo S, Geuna S. Staining methods for normal and regenerative myelin in the nervous system. Pellicciari C, Biggiogera M, editors. Methods Molecular Biology; 2017. 207–218 p. 10.1007/978-1-4939-6788-9_15 28155156

[37] Pothuizen HHJ, Zhang W-N, Jongen-re AL, Feldon ÃJ, Yee BK (2004). Dissociation of function between the dorsal and the ventral hippocampus in spatial learning abilities of the rat : a within-subject , within-task comparison of reference and working spatial memory. Eur J Neurosci.

[38] Moser M, Moser EI (1998). Functional Differentiation in the Hippocampus. Hippocampus..

[39] Tsien JZ, Huerta PT, Tonegawa S (1996). The essential role of hippocampal CA1 NMDA receptor – dependent synaptic plasticity in spatial memory. Cell..

[40] Braak H, Braak E (1991). Neuropathological stageing of Alzheimer-related changes. Acta Neuropathol.

[41] Gomez-Smith M, Karthikeyan S, Jeffers MS, Janik R, Thomason LA, Stefanovic B (2016). A physiological characterization of the Cafeteria diet model of metabolic syndrome in the rat. Physiol Behav.

[42] Pasinetti GM, Eberstein JA (2008). Metabolic syndrome and the role of dietary lifestyles in Alzheimer’s disease. J Neurochem.

[43] Pancani T, Anderson KL, Brewer LD, Kadish I, DeMoll C, Landfield PW (2013). Effect of high-fat diet on metabolic indices, cognition, and neuronal physiology in aging F344 rats. Neurobiol Aging.

[44] Lin B, Hasegawa Y, Takane K, Koibuchi N, Cao C, Kim-Mitsuyama S (2016). High-fat-diet intake enhances cerebral amyloid angiopathy and cognitive impairment in a mouse model of alzheimer’s disease, independently of metabolic disorders. J Am Heart Assoc.

[45] Adolfsson R, Bucht G, Lithner F, Winblad B (1980). Hypoglycemia in Alzheimer’s disease. Acta Med Scand.

[46] Nguyen JCD, Killcross AS, Jenkins TA (2014). Obesity and cognitive decline: role of inflammation and vascular changes. Front Neurosci.

[47] Knight EM, Martins IVA, Gümüsgöz S, Allan SM, Lawrence CB (2014). High-fat diet-induced memory impairment in triple-transgenic Alzheimer’s disease (3xTgAD) mice is independent of changes in amyloid and tau pathology. Neurobiol Aging.

[48] Thériault P, ElAli A, Rivest S (2016). High fat diet exacerbates Alzheimers disease-related pathology in APPswe/PS1 mice. Oncotarget..

[49] Yeh H-H, Li C-H, Huang Y-Y, Huang F-L, Tsay H-J, Huang W-S (2017). Neuroimaging of inflammaion: high fat-induced exacerbation of Alzheimer disease. J Nucl Med.

[50] Aizenstein HJ, Nebes RD, Saxton JA, Price JC, Dekosky ST, Halligan EM (2008). Frequent amyloid deposition without significant cognitive impairment among the elderly. Arch Neurol.

[51] Davis DG, Schmitt RA, Wekstein DR, Markesbery WR (1999). Alzheimer neuropathologic alterations in aged cognitively normal subjeets. J Neuropathol Exp Neurol.

[52] Xiang Z, Haroutunian V, Ho L, Purohit D, Pasinetti GM (2006). Microglia activation in the brain as inflammatory biomarker of Alzheimer’s disease neuropathology and clinical dementia. Dis Markers.

[53] Bilello M, Doshi J, Nabavizadeh SA, Toledo JB, Erus G, Xie SX (2015). Correlating cognitive decline with white matter lesion and brain atrophy MRI measurements in Alzheimer’s disease. J Alzheimers Dis.

[54] Brickman AM, Zahodne LB, Guzman VA, Meier IB, Griffith EY, Provenzano FA (2015). Reconsidering harbingers of dementia: progression of parietal lobe white matter hyperintensities predicts Alzheimer’s disease incidence. Neurobiol Aging.

[55] Reijmer YD, Leemans A, Brundel M, Kappelle LJ, Biessels GJ (2013). Disruption of the cerebral white matter network is related to slowing of information processing speed in patients with type 2 diabetes. Diabetes..

[56] Wang M, Norman JE, Srinivasan VJ, Rutledge JC (2016). Metabolic, inflammatory, and microvascular determinants of white matter disease and cognitive decline. Am J Neurodegener Dis.

[57] Hu X, Li P, Guo Y, Wang H, Leak RK, Chen S (2012). Microglia/macrophage polarization dynamics reveal novel mechanism of injury expansion after focal cerebral ischemia. Stroke..

[58] Kumar A, Alvarez-Croda D-M, Stoica BA, Faden AI, Loanel DJ (2016). Microglial/macrophage polarization dynamics following traumatic brain injury. J Neurotrauma.

[59] Serdar M, Kempe K, Rizazad M, Herz J, Bendix I, Felderhoff-Muser U (2019). Early pro-inflammatory microglia activation after hypoxic-ischemic brain injury in neonatal rats. Front Cell Neurosci.

[60] Zrzavy T, Hoftberger R, Berger T, Rauschka H, Butovsky O, Weiner H (2019). Pro-inflammatory activation of microglia in the brain of patients with sepsis. Neuropathol Appl Neurobiol.

[61] Tang Y, Le W. Differential roles of M1 and M2 microglia in neurodegenerative diseases. Mol Neurobiol. 2015;(April):1–14. 10.1007/s12035-014-9070-5 25598354

[62] Filley CM (2012). White matter dementia. Ther Adv Neurol Disord.

