# Supplementary material for: White matter inflammation and cognitive function in a co-morbid metabolic syndrome and prodromal Alzheimer’s disease rat model
Source: J Neuroinflammation. 2020 Jan 21;17:29. doi: 10.1186/s12974-020-1698-7 (PMC6975033; doi:10.1186/s12974-020-1698-7)
Supplement: Supplementary file 1 — Additional file 1: Figure S1. Arterial blood pressure measured three weeks prior to the diet onset, 6 and 10 weeks on the diet. A) Systolic and B) diastolic blood pressure levels. Figure S2. Morris water maze learning and memory test performed one week prior to the diet onset. A) Latency to reach the platform in the 4-day training phase. B) Time spent in the target quadrant during the probe trial following the learning phase (Pr1) expressed as percent of total time in probe trial. Figure S3. Locomotor activity and anxiety-like behavior in open filed test. (A) Total ambulatory distance for 20 minutes and (B) percentage of time spent in central zone of an open field arena. Figure S4. Pro-inflammatory and anti-inflammatory markers in white matter. 10× photomicrographs of representative iNOS (A) and CD 206 (B) immunolabelled microglial cells in the corpus callosum and internal capsule - boxed regions on coronal brain sections, right hemisphere. Scale bar 100μm. Magnified image of individual iNOS positive cell is inserted on image of Control WT rat in panel A. Images of a positive control for iNOS and CD 206 staining showing positively stained cells in post-stroke striatum is included in respective panels. Figure S5. Myelination of white matter. 10× photomicrographs of representative brain sections stained with Luxol fast blue containing (A) corpus callosum and (B) internal capsule, right hemisphere. Scale bar 100μm. Area coverage by a positive signal (as percentage of a total area of a region) for (C) corpus callosum and (D) internal capsule. Figure S6. Immunohistochemical staining for cerebral amyloid-β deposition. 10× photomicrographs of representative brain sections stained with 4G8 containing hippocampus (CA1 region), cerebral cortex and corpus callosum (periventricular region), right hemisphere. A human tissue sample from a patient with confirmed Alzheimer's disease used as a positive control. Scale bar 100 μm. [file 12974_2020_1698_MOESM1_ESM.docx]

**Supplemental Information**

Proposed title as per Reviewer’s suggestion**:**

**White Matter Inflammation and Cognitive Function in a Co-morbid Metabolic Syndrome and Prodromal Alzheimer’s Disease Rat Model**

**~~Co-Morbidity of Prodromal Alzheimer’s Disease and Metabolic Syndrome in the APP21 Transgenic Rat~~**

Nadezda Ivanova^1*^, Cansu Agca^2^, Yuksel Agca^2^, Earl G. Noble^3^, Shawn Narain Whitehead^1^, David Floyd Cechetto^1^

^1^Department of Anatomy & Cell Biology, Schulich School of Medicine & Dentistry, Western University, London, ON, Canada

^2^Department of Veterinary Pathobiology, University of Missouri College of Veterinary Medicine, Columbia, MO, USA

^3^School of Kinesiology, Western University, London, ON, Canada

*Address correspondence to: Nadezda Ivanova

Department of Anatomy & Cell Biology, Schulich School of Medicine & Dentistry, Medical Sciences Building, Western University, London, ON, N6A 5C1, Canada.

Tel: 519-661-2111 etx.82724

Email address: [nivanov3@uwo.ca](mailto:nivanov3@uwo.ca)

**Animal numbers**

The number of animals used in the blood pressure assessment and behavioral testing presented in Figure S1 through Figure S3 are as follows: Control WT (n=12), Control TG (n=11), HCD WT (n=12), HCD TG (n=11). Animal numbers per experimental group used in immunohistological analysis of myelination presented in Figure S5 are as follows: Control WT (*n* = 4), Control TG (*n* = 4), HCD WT (*n* = 4), HCD TG (*n* = 4).

**Statistical analysis**

Statistical tests used included Two - way ANOVA (Figure S1), RM Two - way ANOVA (Figure S2A) and One - way ANOVA (Figure S2B, Figure S3, Figure S5) which were followed by Tukey’s multiple comparisons test. Values are presented as mean ± standard error of the mean (SEM). A *p* value of ≤ 0.05 was considered statistically significant.

In Figure S2, significance is indicated by * between days 1 and 4 in all groups. In Figure S3, significance is indicated by * for Control WT vs Control TG and HCD TG.

**Abbreviations**

Abbreviations used in the Figure legends are the following: HCD = hypercaloric diet, TG = transgenic, WT = wildtype.

**
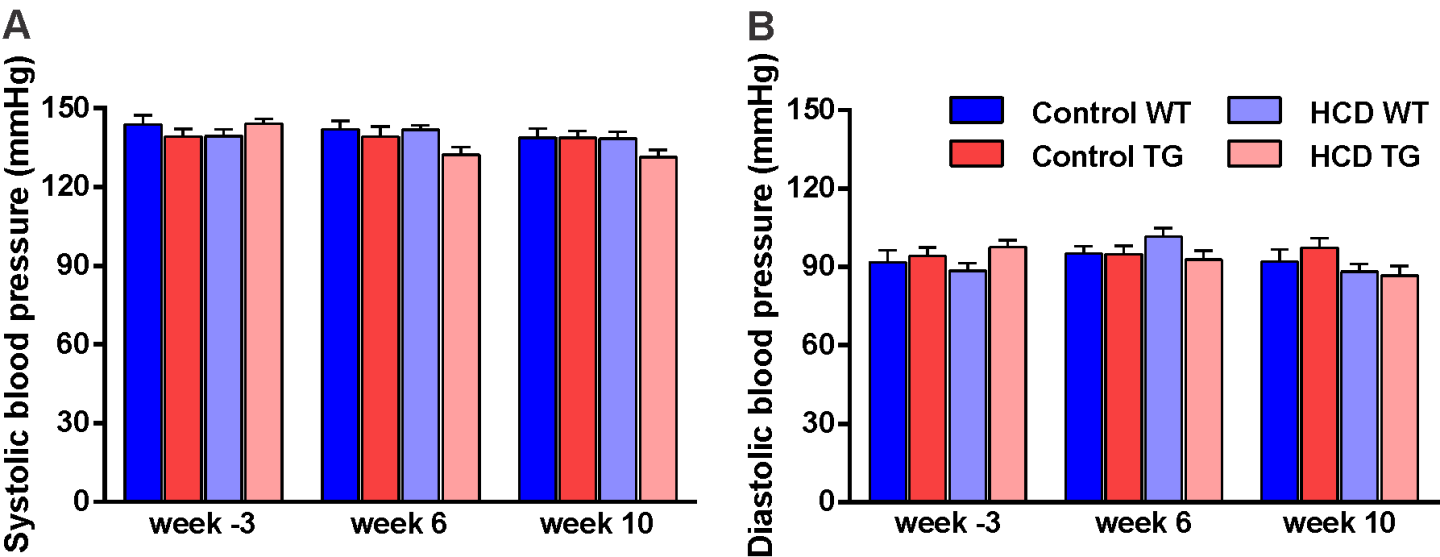
**

**Figure S1. *Arterial blood pressure measured three weeks prior to the diet onset, 6 and 10 weeks on the diet.* (A)** Systolic and **(B)** diastolic blood pressure levels.


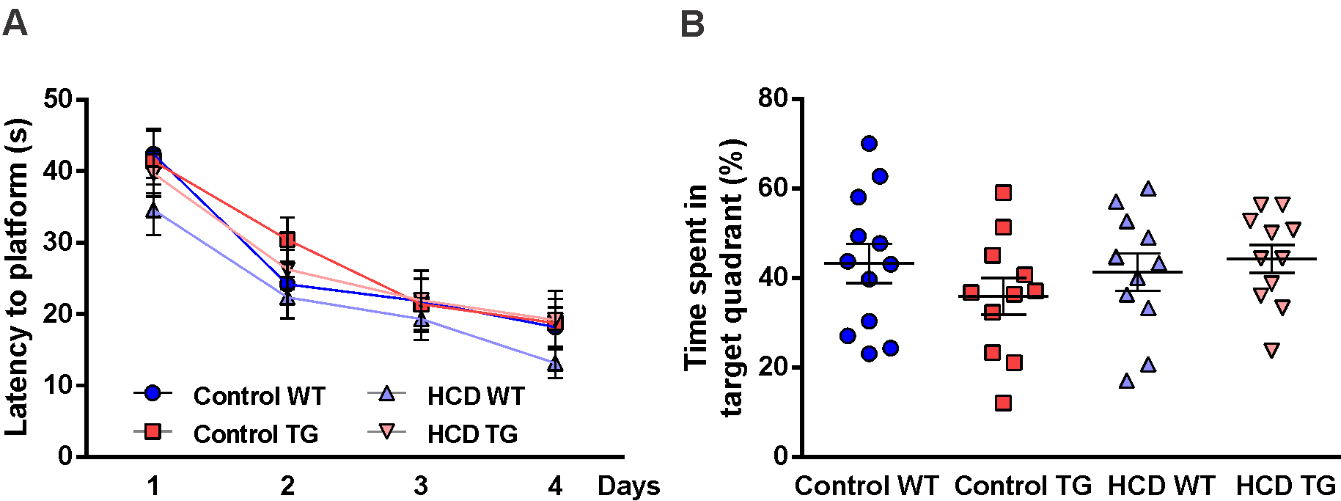


**Figure S2. *Morris water maze learning and memory test performed one week prior to the diet onset.* A)** Latency to reach the platform in the 4-day training phase. **B)** Time spent in the target quadrant during the probe trial following the learning phase (Pr1) expressed as percent of total time in probe trial.


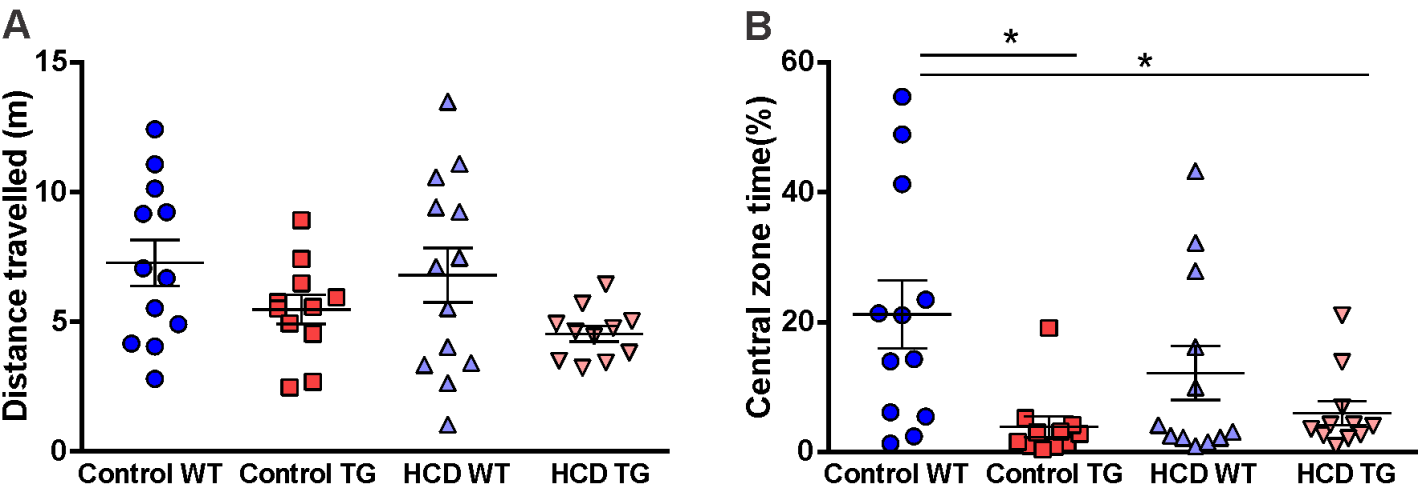


**Figure S3. *Locomotor activity and anxiety-like behavior in open filed test.* (A)** Total ambulatory distance for 20 minutes and **(B)** percentage of time spent in central zone of an open field arena.


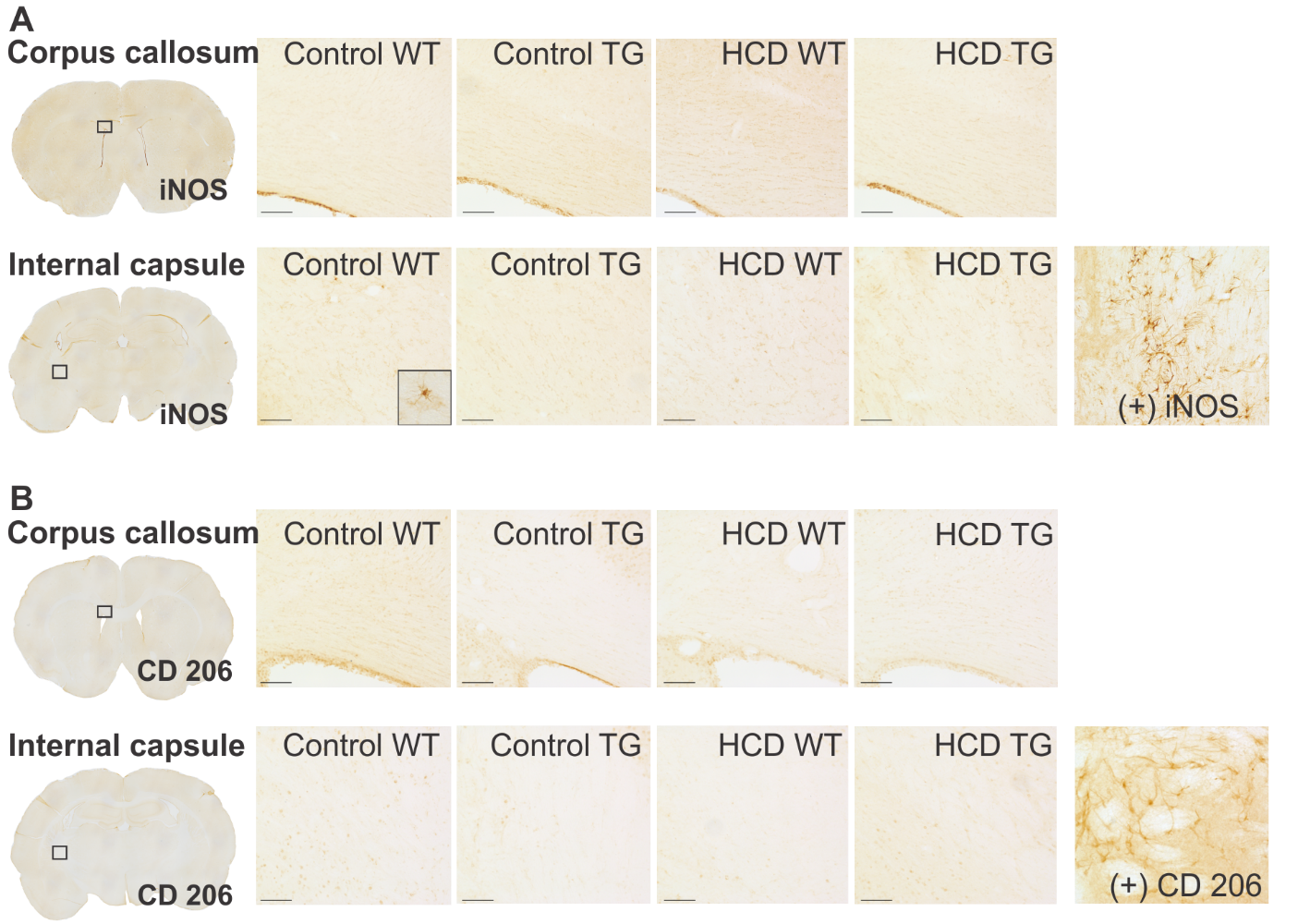


**Figure S4.  *Pro-inflammatory and anti-inflammatory markers in white matter.*** 10x photomicrographs of representative iNOS **(A)** and CD 206 (**B)** immunolabelled microglial cells in the corpus callosum and internal capsule - boxed regions on coronal brain sections, right hemisphere. Scale bar 100µm. Magnified image of individual iNOS positive cell is inserted on image of Control WT rat in panel A. Images of a positive control for iNOS and CD 206 staining showing positively stained cells in post-stroke striatum is included in respective panels.


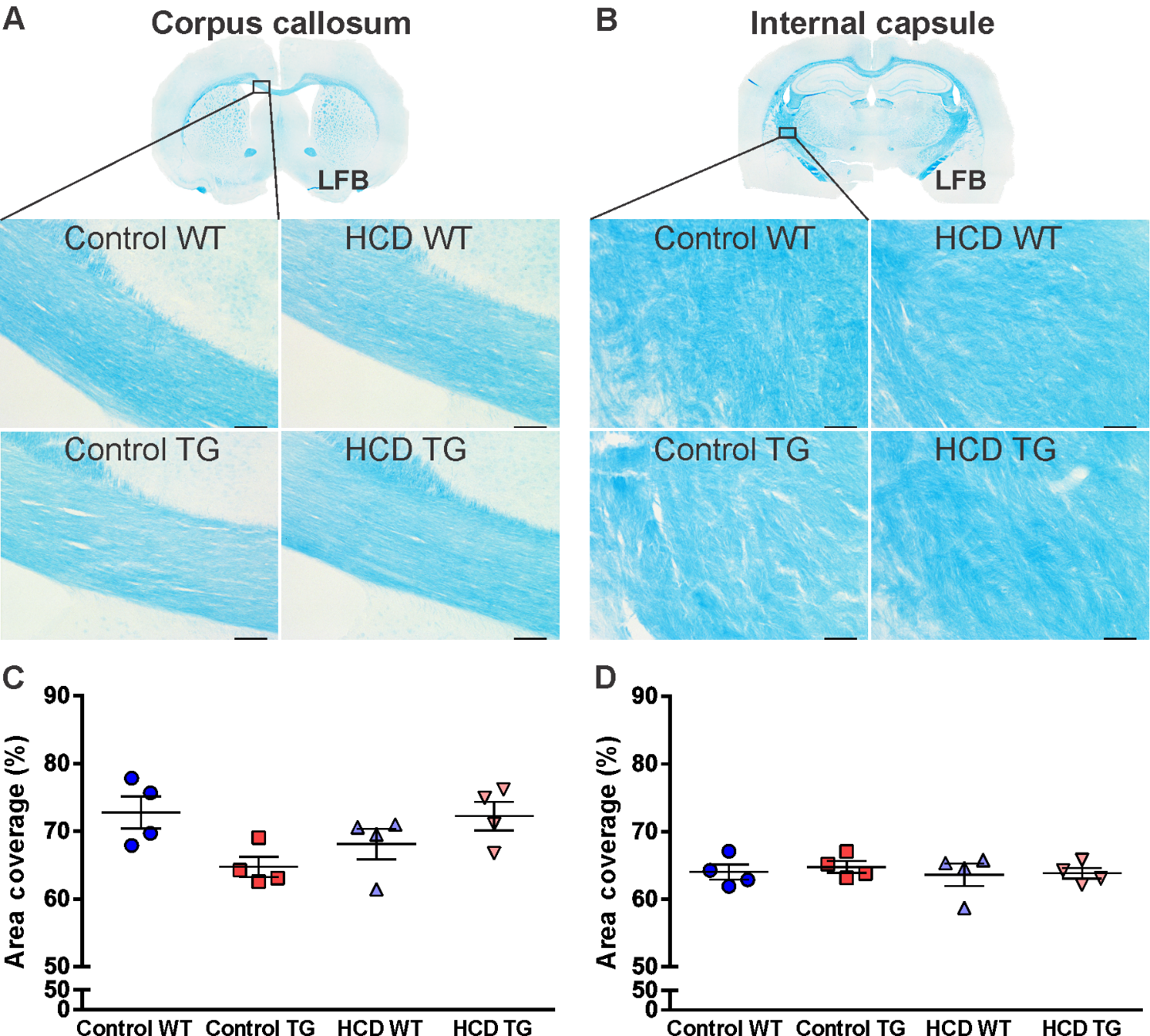


**Figure S5.  *Myelination of white matter.*** 10x photomicrographs of representative brain sections stained with Luxol fast blue containing **(A)** corpus callosum and **(B)** internal capsule, right hemisphere. Scale bar 100µm. Area coverage by a positive signal (as percentage of a total area of a region) for **(C)** corpus callosum and **(D)** internal capsule.

**
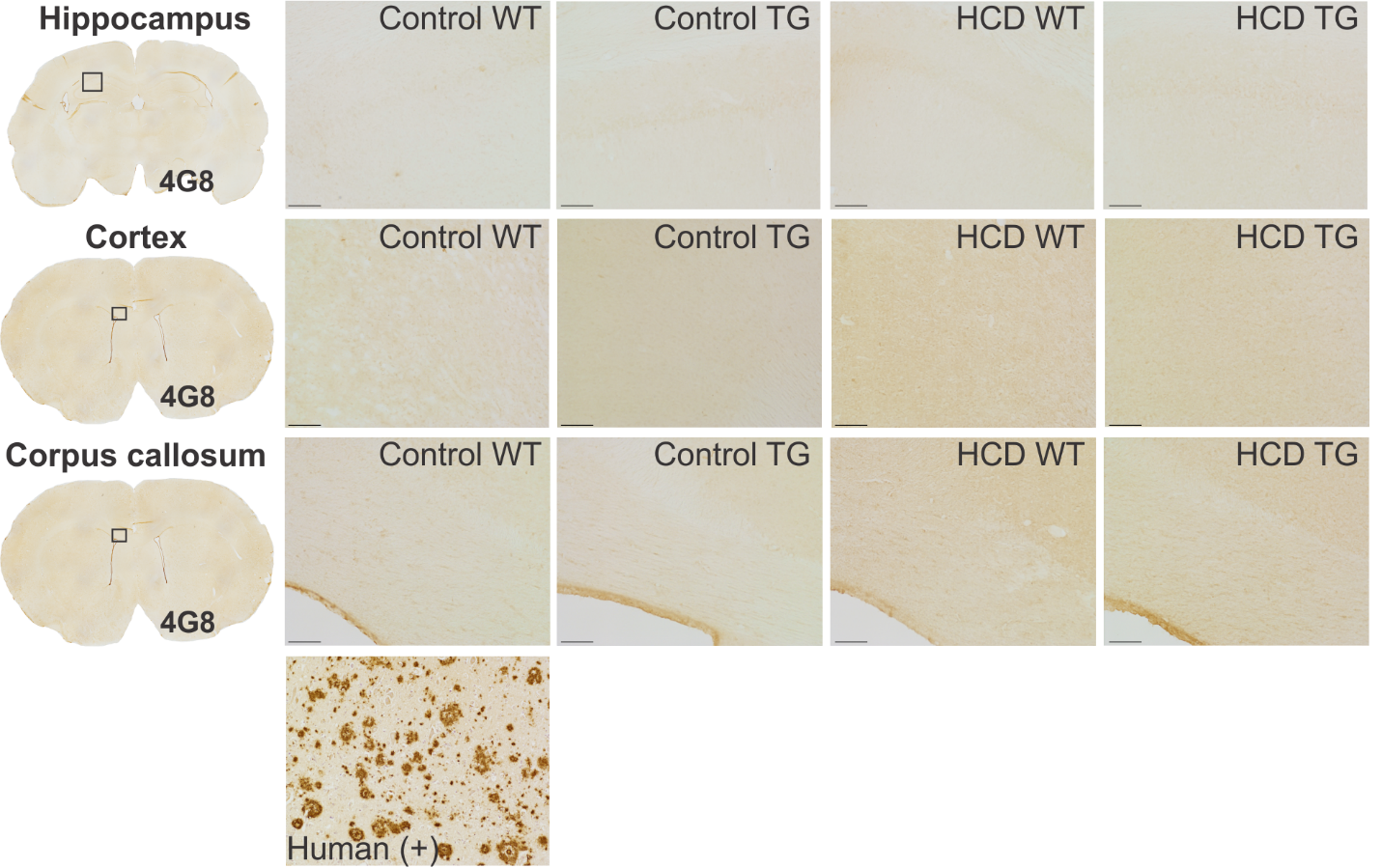
**

**Figure S6. Immunohistochemical staining for cerebral amyloid-β deposition*.*** 10x photomicrographs of representative brain sections stained with 4G8 containing hippocampus (CA1 region), cerebral cortex and corpus callosum (periventricular region), right hemisphere. A human tissue sample from a patient with confirmed Alzheimer disease used as a positive control. Scale bar 100µm.
